# Alpha-synuclein deposition patterns in Alzheimer’s disease: association with cortical amyloid beta and variable tau load

**DOI:** 10.1007/s00401-025-02952-w

**Published:** 2025-10-30

**Authors:** Antonia Neubauer, Doris Weissenbrunner, Susanna Pekrun, Sigrun Roeber, Viktoria Ruf, Paul Feyen, Felix L. Strübing, Jochen Herms

**Affiliations:** 1 Center for Neuropathology and Prion Research, Ludwig Maximilians University of Munich, Munich, Germany; 2 German Center for Neurodegenerative Diseases (DZNE), Munich, Germany; 3 Munich Cluster for Systems Neurology (SyNergy), Munich, Germany

**Keywords:** Alzheimer’s disease, Lewy body disease, Mixed pathology, Alpha-synuclein, Immunohistochemistry, Quantitative neuropathology

## Abstract

Alpha-synuclein (α-syn) deposits are common in around half of the Alzheimer’s disease (AD) cases. While direct and indirect protein interactions are suggested, the relationships between different protein aggregates remain poorly understood. Here, we aimed to characterize α-syn, amyloid beta (Aβ), and tau load distributions of AD patients. Protein deposits were automatically quantified with random forest pixel classifiers in immunohistochemical stains of up to 28 brain regions in 72 brains with advanced AD neuropathological change. α-syn-negative cases were distinguished from amygdala predominant, brainstem predominant, and cortical α-syn-positive cases. Relationships with age, sex, and ApoE genotype were examined. α-syn co-pathology was detected in 60% of AD cases, more frequently, although not significantly, in women. Half of these positive cases presented α-syn deposits in the cortex, around one-third predominantly in the amygdala, and the remaining cases primarily in the brainstem. A high α-syn load in the amygdala was associated with an increased cortical Aβ load. The cortical tau load was increased in the amygdala-predominant α-syn group, but decreased in the brainstem-predominant and cortical α-syn cases in comparison with α-syn-negative cases. ApoE4 was associated with higher hippocampal α-syn and cortical Aβ deposition. Younger age at death was associated with a focally higher Aβ and tau load. AD cases with cortical α-syn deposition tended to have a younger age at death. Here, we show that next to age, sex, and ApoE genotype, the α-syn distribution in AD is related to different Aβ and tau loads. This may have therapeutic relevance for identifying patients who respond to Aβ immunotherapy related to tau burden and underpin the need to define α-syn pathology and distribution in early disease stages.

**Supplementary Information:**

The online version contains supplementary material available at 10.1007/s00401-025-02952-w.

## Introduction

Around 57 million people worldwide are affected by dementia, with Alzheimer’s disease (AD) as the most prevalent neurodegenerative disease [41, 42] and dementia with Lewy bodies (DLB) in the second place [82]. Age and an ApoE4 allele are among the most important risk factors for AD and DLB [12, 13, 24, 44]. Although these neurodegenerative diseases are often described with distinct clinical symptoms and varying neuropathological phenotypes, mixed disease forms are common [39, 63, 77].

The neuropathological hallmarks of AD are amyloid β (Aβ) plaques, neurofibrillary tangles and neuropil threads [42]. Aβ is a fragment of the amyloid precursor protein. According to Thal phases (1–5), Aβ plaques first appear in association cortices and in later stages in the subcortical, brainstem, and cerebellum regions [70]. Tau is a microtubule-associated protein that, in its hyperphosphorylated form, is capable of forming aggregates. These tau deposits  are initially observed in the transentorhinal region (stage I) and progressively appear in limbic and isocortical regions (stage VI) as classified by Braak and Braak [15, 78]. The expansion of tau pathology correlates with cognitive decline [17].

DLB is characterized by alpha-synuclein (α-syn) aggregates in the form of intraneuronal Lewy bodies and Lewy neurites [52]. Physiologically, α-syn is a soluble protein at the presynaptic nerve terminals, participating in vesicular trafficking [19, 74]. Lewy pathology can be classified according to Braak staging, which describes its distribution from the brainstem (stage 1) to the temporal mesocortex and ultimately to the neocortex (stage 6) [16], or consensus criteria by McKeith and colleagues [51]. Five main Lewy body distribution patterns were observed in brain autopsies: olfactory only, amygdala predominant, brainstem predominant, limbic, and neocortical [5, 51].

Around 50% of AD patients present with α-syn co-pathology in addition to Aβ and tau deposits [4, 30, 63, 76]. Alpha-synuclein co-pathology is associated with an accelerated cognitive decline [11, 58, 67]. In AD cases, the α-syn deposits are often described in the amygdala and to a lesser extent in other brain regions such as the brainstem, hippocampus, and neocortex [4, 33, 35, 43, 65, 71, 76]. Recently, increasing attention has been paid to the heterogeneity of α-syn distribution in AD [71]. An amygdala-predominant and a caudo-rostral pattern were distinguished in AD cohorts and suggest an adverse association between amygdala-predominant α-syn co-pathology and AD pathology [14, 30, 49, 62]; however, detailed quantitative analyses of Aβ and tau are lacking.

Different associations have been described between α-syn, Aβ, and tau [66]. Histology and PET imaging studies propose positive correlations between α-syn co-pathology and Aβ and tau deposits in AD [28, 64]. On a molecular level, there is evidence for α-syn inducing hyperphosphorylation and fibrillization of tau [32, 55]. Mouse experiments support the hypothesis of α-syn modulating tau spread [9]. Furthermore, human studies have revealed higher Aβ load in α-syn-positive AD cases [72]. Aβ might lead to α-syn phosphorylation and decreased degradation of α-syn and tau [47, 73]. The extent of interactions occurring in humans has not been fully elucidated yet.

In this study, we combine the analysis of AD with and without α-syn co-pathology with the evaluation of heterogeneity in α-syn deposit distribution for improved patient stratification. According to the observed relationships between α-syn, tau, and Aβ described in the literature, we hypothesized that 1) α-syn co-pathology is associated with a higher tau and Aβ load and 2) different α-syn distribution patterns are associated with divergent tau and Aβ loads. We applied automated immunohistochemical image analysis of α-syn, tau, and Aβ in extensively annotated brain regions in a large cohort of neuropathologically confirmed AD cases. We identified α-syn-negative AD cases, amygdala-predominant, brainstem-predominant, and neocortical α-syn distribution patterns, and compared tau and Aβ load between these groups. Finally, the effects of age, sex, and ApoE genotype were examined.

## Materials and methods

### Human cohort and neuropathological assessment

All brain samples were acquired from the Neurobiobank Munich, including sporadic and genetic cases. The Neurobiobank is based on voluntary donation. Informed consent to use the brains was given by all brain donors when alive or by closest dependents following the patient’s presumed will. Brains were collected respecting the guidelines of the local ethics committee and the Code of Conduct of BrainNet Europe [37]. The use of the material for this project was approved by the Neurobiobank Munich committee. The study was conducted under the principles of the Declaration of Helsinki and in accordance with the local ethics committee. Neuropathological diagnostics were performed by at least two board-certified neuropathologists. The inclusion criteria for this study were 1) a registration of the case in the digital data form with availability of digitized slices with 2) the neuropathological diagnosis of Alzheimer’s disease (AD) and 3) a Braak and Braak stage IV, V, or VI. Furthermore, inclusion criteria also include 4) available data about screening for alpha-synucleinopathy, which is standard in current protocols, and 5) availability of brown diaminobenzidine (DAB) stains in contrast to red alkaline phosphatase (AP) stains, which were used much earlier. These inclusion criteria were applied regardless of additional neuropathological diagnoses or the initial clinical assessment. 72 cases were identified in the Neurobiobank fulfilling these criteria.

In particular, cases in the subgroup αSyn + C with cortically disseminated Lewy pathology also frequently received Lewy body dementia as an additional neuropathological diagnosis (21 of 22 cases) and thus are in a spectrum with a transition to Lewy body pathology with extensive AD-related co-pathology.

**Fig. 1:**
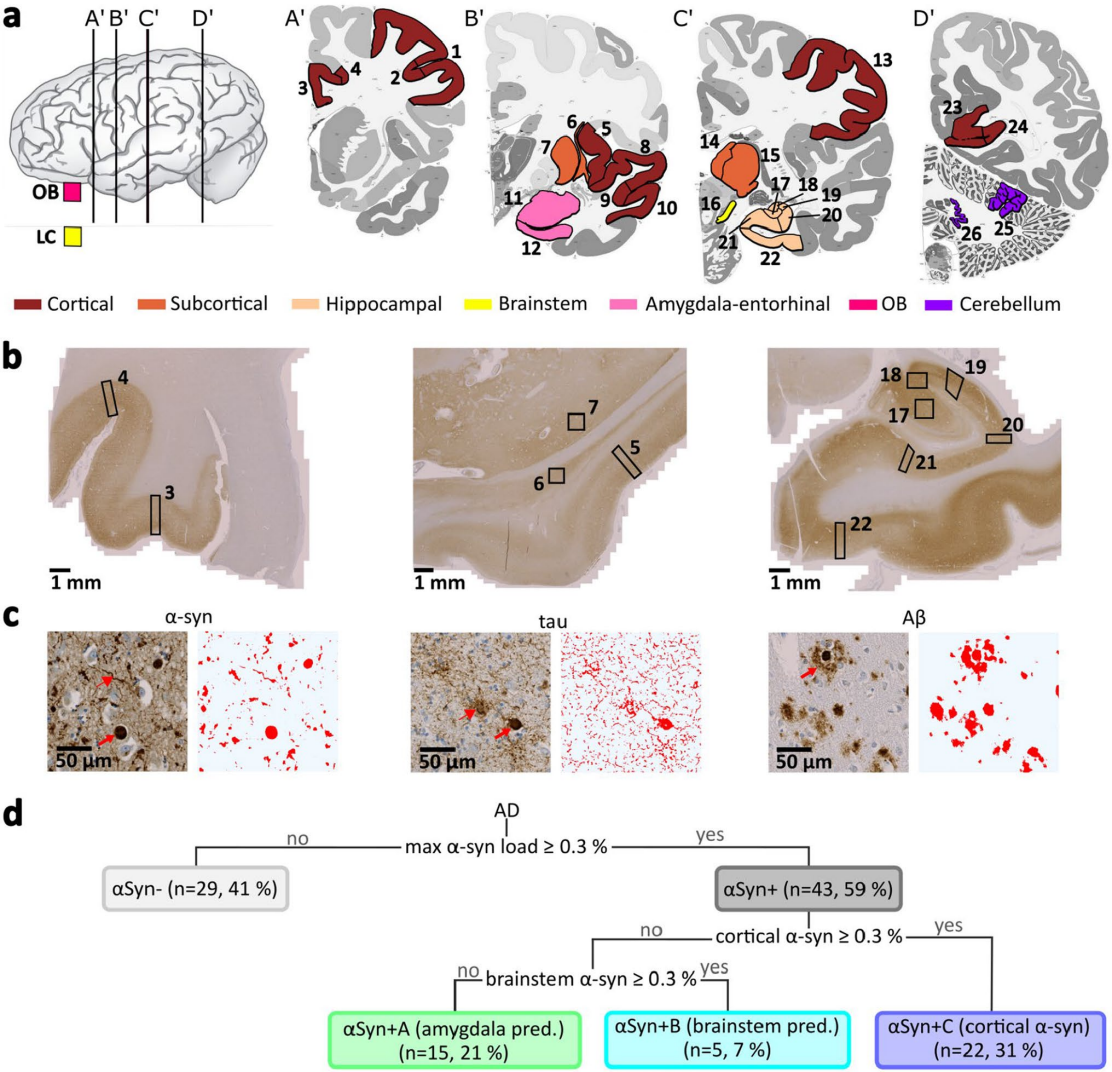
Deposit quantification across brain regions. **a** Overview of analyzed brain regions colored by hypernyms in coronal slices (A’ to D’) in the frame of the gyral Allen Human Brain Atlas (slices A’ to D’: 18, 34, 55, and 83) [3, 22]. **b** α-syn stains of three brain areas with labeled brain regions for further analysis. Blocks from left to right: cingulate gyrus, insula–claustrum–putamen, and hippocampus. Length of bar = 1 mm. **c** Example images with corresponding deposit segmentation for α-syn, tau, and Aβ stains. α-syn: the thick arrow labels a Lewy body; the thin arrow indicates a Lewy neurite. Tau: the thick arrow labels a neurofibrillary tangle bearing neuron; the thin arrow indicates a neuritic plaque. Aβ: the arrow indicates a cored plaque. Length of bar = 50 µm. **d** Schematic overview of α-syn group and subgroup definition by thresholding α-syn-covered areas of all brain regions (max α-syn load), of the mean cortical α-syn load (of cingulate gyrus, superior and medial temporal gyrus, and insula cortex), and of the mean brainstem α-syn load. 1 middle frontal gyrus, 2 sulcus of middle frontal gyrus, 3 cingulate gyrus, 4 sulcus between cingulate and frontal gyrus, 5 insular gyrus, 6 claustrum, 7 putamen, 8 superior temporal gyrus, 9 sulcus between superior and middle temporal gyrus, 10 middle temporal gyrus, 11 amygdala, 12 entorhinal cortex, 13 parietal gyrus, 14 medial thalamus, 15 lateral thalamus, 16 substantia nigra, 17 CA4 of hippocampus, 18 CA3, 19 CA2, 20 CA1, 21 subiculum, 22 parahippocampal gyrus, 23 striate area gyrus, 24 striate area sulcus, 25 cerebellar cortex, 26 dentate nucleus, *AD* Alzheimer’s disease, *LC* locus coeruleus, *max* maximal, *OB* olfactory bulb, *pred.* predominant

For standardized deposit quantification, this study focused on reproducibly identifiable brain regions that are part of the routine diagnostics at the Neurobiobank Munich. In total, 28 gray matter regions were selected, including cortical, subcortical, cerebellar, and brainstem regions (Fig. 1, Table S1). For economic and sustainability reasons, not every brain region was stained for every case. In particular, cases without pathological α-syn in the amygdala and brainstem regions did not necessarily receive α-syn assessment of all other brain regions. To prepare diaminobenzidine stains for light microscopy, the formalin-fixed and paraffin-embedded tissue samples were manually sectioned into 5 µm-thick slices. Further pretreatment and staining were conducted using the automatic system of Ventana BenchMark Ultra (Roche). Sections for α-syn staining were prepared with 80% formic acid for 15 min and boiling pretreatment without further protease-mediated epitope retrieval; sections for AT8 staining received the boiling pretreatment; sections for ßA4 staining were passed through 80% formic acid for 15 min. All sections received treatment with the Cell Conditioning (CC1) Tris-based buffer (Roche). Primary antibodies (Table S2) were used as follows with a Ventana antibody dilution buffer (Roche, #251–018): monoclonal antibody AT8 for phosphorylated tau staining (ThermoFisher, #MN1020; dilution 1:400), monoclonal antibody, clone 4G8, for Aβ (BioLegend, #800,701; dilution 1:5000), and monoclonal α-syn antibody clone 42 (BDTransduction, #610,787; dilution 1:1000). The ultraView Universal DAB Detection Kit (760-500, Roche) was used for detection. A nuclear counterstain was conducted with hematoxylin and bluing reagent (Roche). As the α-syn staining labels also physiological α-synuclein, the subsequent deposit detection tool focuses only on dense positivity like Lewy bodies and Lewy neurites (see “Deposit detection” below). Stains were digitized with a Zeiss Axio Scan Z.1 scanner with a magnification of 20, resulting in a pixel size of 0.22*0.22 µm^2^.

The ApoE status as well as AD-related mutations were obtained through whole genome sequencing. Briefly, DNA was isolated from 1 cm^3^ large tissue cubes taken from fresh-frozen cerebellum using the QIAmp DNA Mini Kit (Qiagen, 51,304). Library preparation was performed with the TruSeq PCR-free genomic DNA library prep kit (Illumina, FC-121–3003) according to the manufacturer’s instructions. Libraries underwent 2 × 150 bp paired-end sequencing on an Illumina NovaSeq machine until a minimum depth of 35X was reached. Alignment and variant calling were performed using a Snakemake pipeline incorporating the GATK best practices. After FastQC and adapter trimming, alignment to the hs1/T2T genome assembly (chm13v2.0) was performed with BWA-MEM2. Variant calling, recalibration, and joint genotyping were done using GATK version 4.0. Subsequently, the APOE genotype was defined by concatenating the APOE-defining variants (rsID/hs1 coordinates: rs429358/chr19:47,733,380; rs7412/chr19:47,733,518).

### Image analysis

The region annotation was conducted manually in Qupath (version 0.5.1) [7] in α-syn stains where available and tau stains as a second choice. The regions were labeled following a protocol to reproducibly set the location and size of the annotations (Supplementary Table S1). In cases with staining artifacts or large blood vessels, the nearest appropriate region was selected in accordance with the protocol. Samples with substantial artifacts or lacking clear orientation to define the region of interest were not included in further analysis. For substantia nigra and locus coeruleus, the annotations avoid pigmented neurons to prevent false positive pixels in the subsequent analysis. The areas were selected to be as representative as possible of the extent of the deposits. Two squares were chosen for substantia nigra and locus coeruleus, respectively, to get the mean as a more robust result. Comparing the deposit covered areas of the first and second annotations with a Wilcoxon signed-rank test, there was no significant difference, suggesting that the process is relatively reproducible (Fig. S1).

Region annotations were transferred from α-syn stains to Aβ and tau stains with non-rigid registration by Deeperhistreg in Python (Python version 3.10.12) [79–81]. For this co-registration, the whole slide images were downsampled by a factor of 30 to reduce the computing load. All region annotations were visually inspected after transfer and upsampling and manually corrected if necessary.

### Deposit detection

The annotated regions were divided into tiles of 4096*4096 pixels (900*900 µm^2^) to reach a reasonable computing capacity. The preprocessing of the tiles included color deconvolution to extract the brown diaminobenzidine signal and conversion to grayscale images, implemented in Python (Python version 3.10.12). These preprocessed images were then classified pixel-wise with a random forest pixel classifier trained with ilastik (version: ilastik-1.4.0.post1-Linux) for each staining (α-syn, Aβ, tau), separately. The models were trained with ten images from different brains and regions with variable deposit load. The α-syn model was optimized to detect dense deposits, mainly Lewy bodies and distinct Lewy neurites, while not labeling physiological synaptic α-syn staining. A threshold of 0.7 was chosen for all random forest classifier models. The output of the random forest pixel classifiers is a pixel-wise binary segmentation of deposits. The proportion of the positively stained area relative to the total tile area is called *covered area* or *load* interchangeably. In a subsequent step, the models were tested using ten independent images from different subjects and regions and were inspected individually. For additional validation, an individual random forest classifier model was created in ilastik for each testing image to gain a reference standard. The results of the previously trained models were compared to these references and evaluated in terms of how many pixels were classified correctly (prediction accuracy) and how close the values of the absolute covered area matched the covered area in the reference independently from the exact localization of the pixels (area accuracy) (Fig. S2).

### Definition of α-syn groups and subgroups in AD

Alzheimer’s disease patients are heterogeneous regarding their α-syn load. The simplest distinguishing criterion is α-syn deposit negative (*αSyn−*) vs. positive (*αSyn* +). Since the α-syn extent represents a smooth transition and might vary in some borderline cases, we defined a threshold of ≥ 0.3% α-syn-covered area in the individually most affected brain region to label a case as αSyn + (Fig. 1). As a minimum requirement, all cases assigned αSyn− needed to have at least an α-syn staining of the amygdala region, as this is one of the most affected brain areas by α-syn in AD. However, as described before [5, 51], different α-syn distribution patterns exist, with a focus on brainstem-, cortical-, and amygdala-predominant forms. To identify these patterns, the threshold of ≥ 0.3% α-syn-covered area was also applied to the mean of the most affected cortical regions (cingulate gyrus, superior and medial temporal gyrus, and insula cortex) and the brainstem (value of substantia nigra or locus coeruleus or mean of both if they were available). Based on these thresholds, the αSyn + group was further divided into three subgroups, namely *αSyn* + *A*, with an amygdala-predominant α-syn deposition, *αSyn* + *B*, with a brainstem predominant α-syn load, and *αSyn* + *C*, with cortical α-syn deposits.

### Statistical analysis

Epidemiological data between α-syn distribution groups were compared with a Mann–Whitney *U* test or Kruskal–Wallis test for continuous data, and a Chi-squared test for categorical data.

α-syn, Aβ, and tau loads of groups and subgroups of AD patients were compared with multiple linear regression to control for age and sex. Five clusters of brain regions were defined to condense the large number of regions, namely, cortical, subcortical, hippocampal, brainstem, and amygdala–entorhinal cluster (Fig. 1), leading to the following formula for each region cluster, respectively:

$${\text{Covered area }}\sim \, \left( {{\text{sub}} - } \right){\text{group name }} + {\text{ region name }} + {\text{ sex }} + {\text{ age}}{.}$$

“Covered area” is the covered area/load of α-syn, Aβ, or tau. “(Sub-)group name” represents the name of the α-syn group or subgroup defined by thresholds (see “Definition of α-syn groups and subgroups in AD” and Fig. 1). Groups/subgroups were compared pairwise. As region clusters were the input data, “region name” is a fixed effect for every individual brain region. Sex and age were added as further control parameters. As control analyses, multiple linear regression was repeated without age and sex correction, or with ApoE4 carriage as an additional control parameter, alongside age, sex, and region name. Additionally, linear mixed-effects models were applied, incorporating random effects for each subject (1 | subject ID) into the above formula.

To examine the association of α-syn load with age, sex, and ApoE status, we defined age groups (< 65 years at death (< 65), 65 to < 75 years (65–75), 75 years or older (≥ 75)) and divided the AD patients with available ApoE status into ApoE4 carriers, defined as at least one ApoE4 allele, vs. no ApoE4. Subsequently, we applied multiple linear regression within each region cluster, controlling for the specific region names. Additional analyses were conducted, controlling for age and sex. These analyses were repeated for tau and Aβ load in parallel.

All *p*-values were corrected for false discovery rate (FDR correction in R) for each analysis, respectively. Statistical tests were conducted with R (R version 4.1.2). The significance level was set to *p < 0.05, **p < 0.01, and ***p < 0.001. Plots were created with Python (Python version 3.10.12). Color plotting on brain atlas images was conducted with Python in combination with Inkscape (Inkscape version 1.4), and the code was made publicly available on GitHub (https://github.com/cor2ni/2D_brain_plot).

## Results

To analyze the association of α-syn load and distribution with Aβ and tau pathology in AD, we analyzed immunohistochemical stains of up to 28 brain regions per case in 72 AD patients (Table 1). Due to a recruitment bias in voluntary brain donation, the exact ratios are exemplary and cannot be directly transferred to a new population. In particular, a shift toward younger and family-related cases is to be expected, and the following characteristics help to estimate the bias for conclusions. The cohort had a mean age at death of 72.8 years (± 11.5 years standard deviation). 56% of the subjects were female. Most of the cases had a Braak and Braak stage VI and a Thal phase 5, corresponding to an advanced stage of AD. For 66 cases, information about the ApoE status was available, revealing at least one ApoE4 allele in 58% of the subjects. An AD-related mutation (APP/PSEN1/PSEN2/TREM2) was reported in 19 out of 72 patients (26%) (Table S3).

**Table 1:** Demographic, clinical and neuropathological overview of α-syn groups in Alzheimer's disease

	Available n	All	αSyn−	αSyn +	Statistic, *p*-value
n (%)	72	72 (100%)	29 (40%)	43 (60%)	
Clinical diagnosis AD: n (%) PD: n (%)	72	AD: 42 (58%) PD: 6 (8%)	AD: 15 (52%) PD: 0 (0%)	AD: 27 (63%) PD: 6 (14%)	
Sex (female:male)	72	40:32	13:16	27:16	*χ*2 = 1.6, *p* = 0.21
Age at onset [years]	65	61.0 ± 12.2	62.5 ± 12.7	60.2 ± 11.8	*U* = 429, *p* = 0.4
Disease duration [years]	65	11.0 ± 5.9	10.4 ± 4.9	11.4 ± 6.4	*U* = 543, *p* = 0.5
Age at death [years]	71	72.8 ± 11.5	73.7 ± 10.5	72.2 ± 12.0	*U* = 561, *p* = 0.6
Braak and Braak (IV:V:VI)	72	8:14:50	1:7:21	7:7:29	*χ*2 = 3.2, *p* = 0.21
Thal phase (3:4:5)	69^a^	2:7:60	1:2:25	1:5:35	*χ*2 = 3.4, *p* = 0.5
TDP43 (neg:pos)	49	25:24	14:7	11:17	*χ*2 = 2.6, *p* = 0.11
ApoE4 allele (neg:pos)	66	28:38	15:12	13:26	*χ*2 = 2.4, *p* = 0.12

Age at onset/death and disease duration are presented as mean ± first standard deviation; *U* two-sided Mann–Whitney U test; *χ 2* Chi-squared test

*AD* Alzheimer’s disease, *PD* Parkinson’s disease, *neg* negative, *pos* positive

^a^The three missing cases have a Thal phase ≥ 3

**Fig. 2:**
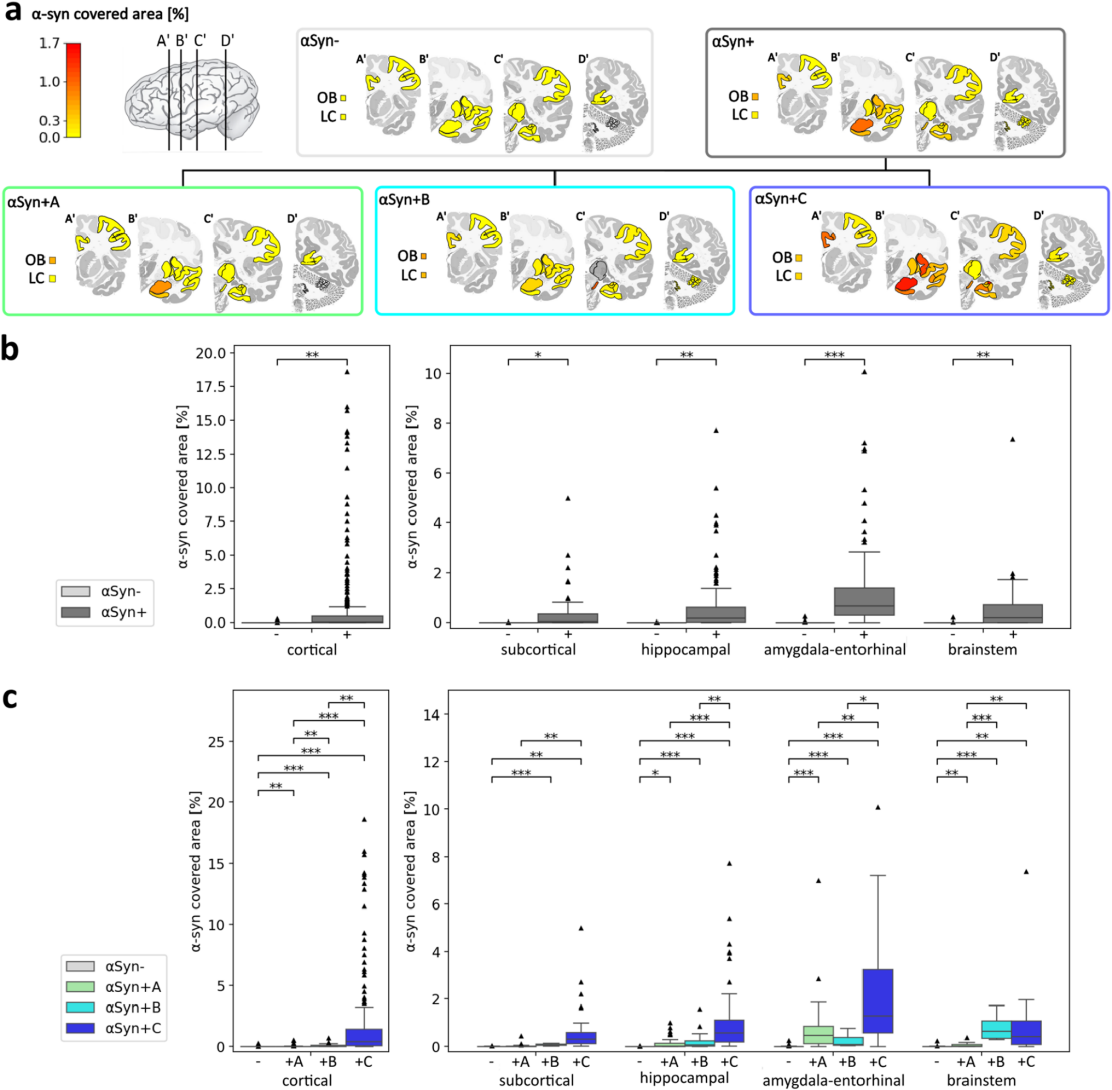
Alpha-Synuclein (α-syn) load and distribution in Alzheimer’s disease cases. **a** Median α-syn-covered area of each α-syn distribution group and subgroup. **b** Comparison of the α-syn-covered area between αSyn- and αSyn + groups. **c** Comparison of the α-syn-covered area between αSyn- and αSyn + A (amygdala predominant), αSyn + B (brainstem predominant), and αSyn + C (cortical) α-syn-positive subgroups. Statistics in **b** and **c** were calculated with multiple linear regression across region clusters, controlling for region names, age, and sex, and false discovery rate correction. Boxplots complemented with scatter dots for female and male patients are available in the supplementary Fig. S6. *LC* locus coeruleus, *OB* olfactory bulb

The deposit covered area was automatically quantified by random forest pixel classifiers in 1016 regions in α-syn stains, 1292 regions in tau stains, and 1098 regions in Aβ stains. By thresholding, AD patients were assigned to αSyn−, comprising 41% of the cases, and αSyn + , including 59% of the cases (Figs. 1 and 2). Thereby, α-Syn positivity was comparably common in genetic and sporadic AD cases (Table S3). The αSyn + group was further divided into three α-syn distribution patterns (Figs. 1 and 2, Table 2, Table S8): αSyn + A, comprising around one-third of the α-syn-positive cases with an almost exclusive amygdala–entorhinal α-syn load; αSyn + B, including around 12% of the α-syn-positive cases and characterized by a high brainstem α-syn load without cortical spread and a low amygdala involvement; αSyn + C, comprising around half of the α-syn-positive cases and presenting with at least focal cortical α-syn deposits together with the highest amygdala–entorhinal and a relatively high brainstem α-syn load. Although not significantly different, genetic cases and cases with the youngest age at symptom onset were predominantly assigned to αSyn− and αSyn + C groups, while the αSyn + A group was more common in sporadic cases (Fig. S3, Table S3). All groups and subgroups were evaluated regarding their Aβ and tau load, revealing distinct loads in different brain regions.

**Table 2:** Demographic, clinical, and neuropathological overview of α-syn distribution subgroups

	Avail. n	αSyn−	αSyn + A (amygdala pred.)	αSyn + B (brainstem pred.)	αSyn + C (cortical α-syn)	Statistic, *p*-value
n (%)	71	29 (41%)	15 (21%)	5 (7%)	22 (31%)	
Clinical diagnosis AD: n (%) PD: n (%)	71	AD: 15 (52%) PD: 0 (0%)	AD: 10 (67%) PD: 0 (0%)	AD: 2 (40%) PD: 2 (40%)	AD: 14 (64%) PD: 4 (18%)	
Sex (female:male)	39:32	13:16	11:4	2:3	13:9	*χ*2 = 3.9, *p* = 0.28
Age at onset [years]	64	62.5 ± 12.7	64.1 ± 8.7	64.3 ± 6.9	56.5 ± 13.6	*K* = 2.81, *p* = 0.4
Disease duration [years]	64	10.4 ± 4.9	12.4 ± 6.0	11.2 ± 8.5	10.8 ± 6.1	*K* = 1.36, *P* = 0.71
Age at death	70	73.7 ± 10.5	76.5 ± 9.2	76.6 ± 5.6	68.5 ± 13.6	*K* = 4.07, *p* = 0.25
Braak and Braak (IV:V:VI)	71	1:7:21	0:1:14	2:2:1	5:4:13	*χ*2 = 15.5, *p* = **0.016**^b^
Thal phase (3:4:5)	68^a^	1:2:25	0:0:15	0:2:3	1:3:16	*χ*2 = 14.6, *p* = 0.27
TDP43 (neg:pos)	49	14:7	5:6	3:2	3:9	*χ*2 = 5.6, *p* = 0.13
ApoE4 allele (neg:pos)	65	15:12	6:7	1:4	5:15	*χ*2 = 5.5, *p* = 0.14

Significant *p*-values are labeled in bold

Age at onset/death and disease duration are presented as mean ± first standard deviation

*K* Kruskal–Wallis test, *χ 2* Chi-squared test, *AD* Alzheimer’s disease*, PD* Parkinson’s disease*,* a*vail* available, *neg* negative, *pos* positive*, pred* predominant

^a^The three missing cases have a Thal phase ≥ 3

^b^Braak and Braak staging was significantly different between αSyn− and αSyn + B (*χ*2 = 8.7, *p* = 0.013), and αSyn + A and αSyn + B (*χ*2 = 11.5, *p* = 0.003)

### α-syn load and distribution in AD

The αSyn− and αSyn + cases showed a comparable distribution of age at clinical onset (two-sided Mann–Whitney *U* test: *p* = 0.4), disease duration (*p* = 0.5), and age at death (*p* = 0.6) (Table 1 and Fig. S3). In the αSyn + group, there were comparatively more female than male subjects, while the αSyn- group had a slight male predominance, although the difference was not significant (*p* = 0.21). In total, 58% of the patients had a clinical diagnosis of Alzheimer’s disease during their lifetime (Fig. S4). While αSyn− cases were mostly diagnosed with AD (52%), not further specified dementia (24%), and frontotemporal dementia (21%), αSyn + cases were commonly diagnosed with AD (63%), Parkinson’s disease (14%), not further specified dementia (9%), and dementia with Lewy bodies (7%). There was no significant difference regarding the Braak and Braak staging (*p* = 0.21) or Thal phase (*p* = 0.5) between groups. There were proportionally more TDP43-positive cases in the αSyn + group; however, not significantly (*p* = 0.11). There were also more cases carrying at least one ApoE4 allele in proportion to non-carriers in the αSyn + group than in the αSyn− group, but also not significantly (*p* = 0.12). Thus, there might be a female sex, TDP43, and ApoE4 bias in the α-syn-positive group, even without reaching significance. However, it is not clear if this association is causally related or a limitation of the available cohort.

By definition, the αSyn + cases showed a higher α-syn load than αSyn− cases. Performing multiple linear regression correcting for the specific region name, age, and sex, there was a significant difference between αSyn + and αSyn− cases in cortical, subcortical, hippocampal, amygdala–entorhinal, and brainstem region clusters (Table 3), confirming the split into these two groups. The αSyn + cases showed the highest median α-syn load in the amygdala–entorhinal area, followed by the brainstem and hippocampal region, and low coverage in subcortical areas (Fig. 2). The α-syn load in cortical regions was low in the median, but showed a large variability and thereby reached the highest values of covered area in single subjects. These findings suggest a region-dependent predestination for α-syn deposits in AD with a special focus on the amygdala in many cases, as well as a broad inter-patient variability.

**Table 3:** Comparison of the α-syn-covered area between αSyn + vs. αSyn− cases with multiple linear regression controlling for age and sex and correction for false discovery rate

Region cluster	n (αSyn-)	n (αSyn +)	Median [IQR] [%] of αSyn- cases	Median [IQR] [%] of αSyn + cases	β, *p*-value (age, sex corrected)
Cortical	73	348	0.001 [0.0004; 0.003]	0.09 [0.008; 0.48]	*β* = 0.009, *p* = **0.004**
Subcortical	22	97	0.001 [0.0001; 0.003]	0.06 [0.015; 0.35]	*β* = 0.003, *p* = **0.026**
Hippocampal	30	176	0.0006 [0.0002; 0.002]	0.17 [0.017; 0.61]	*β* = 0.005, *p* = **0.004**
Amygdala–entorhinal	54	70	0.0013 [0.0002; 0.006]	**0.67** [0.30; 1.4]	*β* = 0.013, *p* **< 0.001**
Brainstem	36	72	0.0025 [0.0007; 0.006]	0.19 [0.014; 0.72]	*β* = 0.005, *p* = **0.004**

Significant p-values and the highest median α-syn-covered area of the αSyn + group are labeled in bold

*ID* subject ID, *IQR* interquartile range

As additional control analyses, we conducted multiple linear regression without correction for sex and age or with ApoE4 as an additional control factor. Both models showed significantly higher α-syn load in the αSyn + group in all region clusters. Furthermore, applying linear mixed-effects models with correction for age, sex, and a random factor for subject ID, only the difference in the amygdala–entorhinal region remained significant, indicating a strong difference in the amygdala (Table S4). Comparing the α-syn load of αSyn + with αSyn− cases in 28 brain regions separately under correction of age and sex, there was a significantly higher α-syn-covered area in the substantia nigra (*p* = 0.005), amygdala (*p* = 0.005), entorhinal cortex (*p* = 0.023), and olfactory bulb (*p* = 0.023), suggesting these regions as a focus of α-syn co-pathology in AD (Table S5). Other brain regions, e.g., the hippocampus and insula cortex, are also affected. However, probably due to the small absolute numbers, the *p*-values were not statistically significant for other brain regions.

Comparing α-syn-positive subgroups with αSyn− cases, all subgroups showed a comparable age at clinical onset, disease durations, and age at death (Table 2 and Fig. S3). Most of the αSyn + A (67%) and αSyn + C (64%) cases were clinically diagnosed with Alzheimer’s disease (Fig. S4), suggesting AD typical symptoms even with Lewy co-pathology. In contrast, two out of five (40%) αSyn + B cases were diagnosed with Alzheimer’s disease and 40% with Parkinson’s disease, indicating relatively dominant parkinsonian symptoms in the αSyn + B subgroup. Regarding sex distribution, there was a female preponderance in αSyn + A; however, it did not reach significance (*p* = 0.28). There was a trend toward younger age at death in αSyn + C, with a mean age of 68.5 years (± 13.6 years standard deviation) in comparison with 76.5 ± 9.2 years in αSyn + A, 76.6 ± 5.6 years in αSyn + B, and 73.7 ± 10.5 years in αSyn- cases. Although this finding did not reach significance (*p* = 0.25) and there was broad variability between cases, this observation suggests a negative association between cortically spread α-syn pathology in AD and survival. The Braak and Braak staging distribution was shifted toward lower Braak and Braak stages in αSyn + B, which reached significance when comparing αSyn− and αSyn + B (*p* = 0.013), as well as between αSyn + A and αSyn + B (*p* = 0.003). There was no significant difference regarding the Thal phases (*p* = 0.27). Where TDP43 information was available, two-thirds of the αSyn− cases were also TDP43 negative, while three-quarters of the αSyn + C subgroup were TDP43 positive. Performing a Chi-squared test over these groups, there was also no significant difference (*p* = 0.13). Regarding the presence or absence of the ApoE4 allele, 75% of the αSyn + C cases had at least one ApoE4 allele, while it was more balanced in αSyn− and αSyn + A cases, although these group comparisons did not reach significance in a Chi-squared test (*p* = 0.14).

To confirm that the α-syn distribution subgroups vary in their α-syn distributions, we applied multiple linear regression controlling for specific region names, sex, and age. Detailed results are presented in Fig. 2 and Tables S8 and S11. In pairwise tests, all groups are significantly different from each other in their α-syn load across cortical regions, with the highest α-syn load in αSyn + C and, after a large gap, αSyn + B in second place. αSyn + C and, to a lesser extent, αSyn + B show significantly higher subcortical α-syn load than αSyn− cases. αSyn + C significantly shows the highest hippocampal and amygdala–entorhinal α-syn load, much higher than the actual amygdala–entorhinal predominant α-syn subgroup αSyn + A. αSyn + B and αSyn + C show higher brainstem α-syn loads than subgroup αSyn + A. Within αSyn + A, the highest α-syn load is in the amygdala and lower in other brain regions. αSyn + B shows the highest α-syn levels in the brainstem with low values in other brain regions, affirming its definition. Interestingly, αSyn + C manifests with an α-syn amygdala predominance next to high deposit loads in some cortical regions, and often a lower but still high amount in other brain regions. The high deposit load in the amygdala in αSyn + C suggests a general α-syn sensitivity of the amygdala in AD, independent of the exact α-syn distribution type. In total, the identified distribution patterns propose the presence of distinct pathological α-syn accumulation features with overlaps, e.g., in the amygdala.

### Tau load in relation to α-syn distribution

**Fig. 3:**
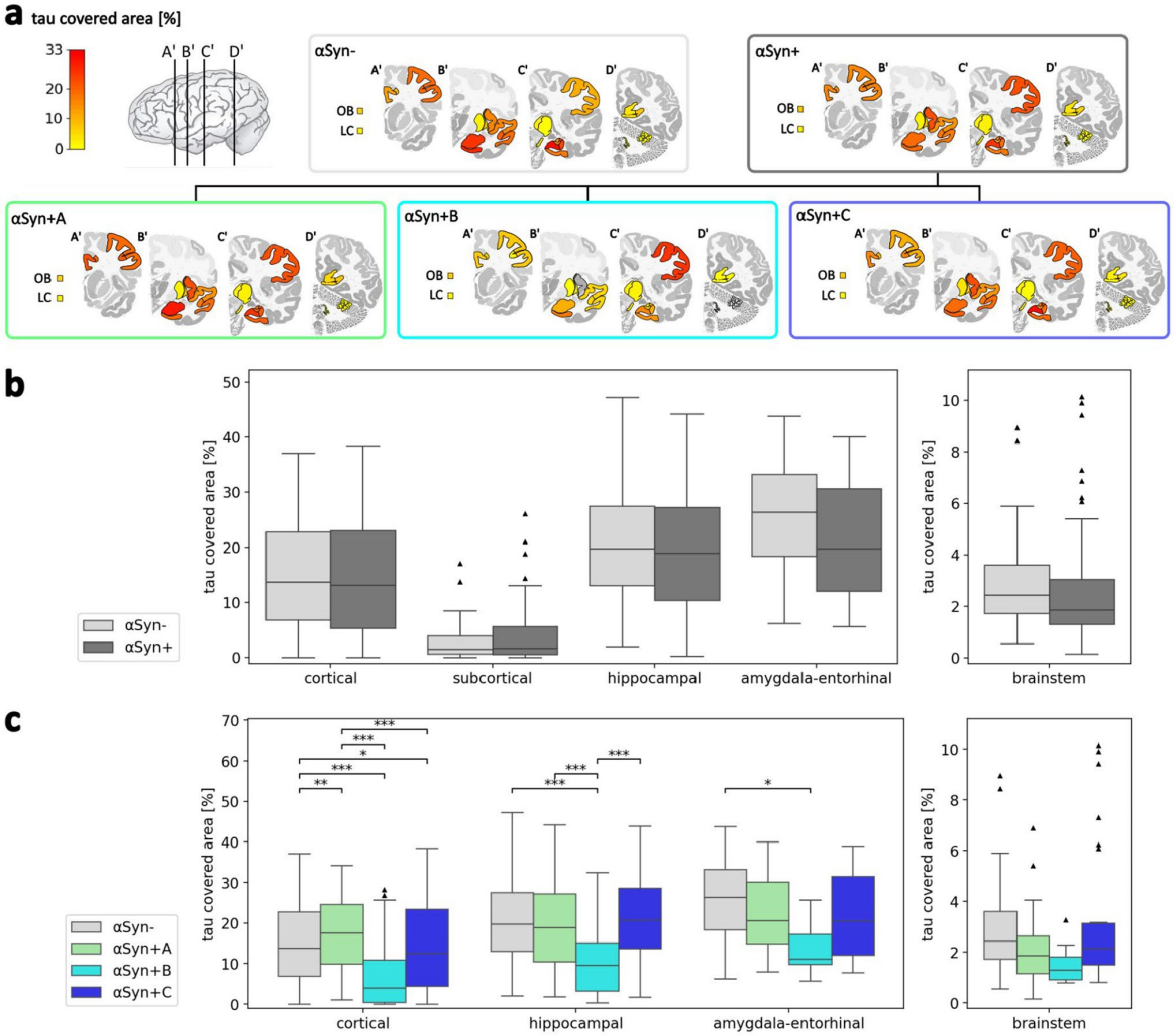
Tau load and distribution in Alzheimer’s disease. **a** Median tau-covered area of each α-syn distribution group and subgroup. **b** Comparison of the tau-covered area between αSyn- and αSyn + groups. **c** Comparison of the tau-covered area between αSyn− and αSyn + A (amygdala predominant), αSyn + B (brainstem predominant), αSyn + C (cortical) α-syn-positive subgroups. Statistics in **b** and **c** were calculated with multiple linear regression across region clusters, controlling for region names, age, and sex, and false discovery rate correction. Boxplots complemented with scatter dots for female and male patients are available in the supplementary Fig. S7. *LC* locus coeruleus, *OB* olfactory bulb

According to the inclusion criteria of Braak and Braak stage ≥ IV, all AD cases showed marked tau pathology. The most affected area was the amygdala–entorhinal region, followed by the hippocampal region and the cortical region in third place (Table 4). There was a low tau-covered area in the brainstem and subcortical areas. To examine potential associations between tau and α-syn loads, we compared the tau-covered area of αSyn- vs. αSyn + cases with multiple linear regression, correcting for the specific region name, age, and sex. Interestingly, there was no significant effect of α-syn presence on tau load in any brain region cluster (Fig. 3, Table 4), suggesting independent accumulation of α-syn and (AT8-) hyperphosphorylated tau.

**Table 4:** Comparison of the tau-covered area between αSyn + vs. αSyn− cases with multiple linear regression controlling for age and sex and correction for false discovery rate

Region cluster	n (αSyn−)	n (αSyn +)	Median [IQR] [%] of αSyn- cases	Median [IQR] [%] of αSyn + cases	β, p-value (age, sex corrected)
Cortical	216	355	14.4 [7.7; 23]	13.2 [5.3; 23.1]	*β* = − 0.012, *p* = 0.19
Subcortical	39	72	1.5 [0.6; 4]	1.7 [0.6; 5.6]	*β* = − 0.003, *p* = 0.72
Hippocampal	133	222	19.8 [13.1; 27.5]	18.9 [10.4; 27.3]	*β* = − 0.017, *p* = 0.19
Amygdala–entorhinal	42	63	**26.3** [18.3; 33]	**19.8** [12; 30.6]	*β* = − 0.043, *p* = 0.12
Brainstem	35	55	2.4 [1.7; 3.6]	1.9 [01.3; 3]	*β* = − 0.002, *p* = 0.72

The highest median tau covered areas of αSyn + and αSyn−- groups were labeled in bold

*ID* subject ID, *IQR* interquartile range

As additional control analyses, we conducted multiple linear regression without correction for sex and age or with additional correction for ApoE4. Following the previous analysis, there was no significant difference regarding the tau load between αSyn− and αSyn + groups in all region clusters. Furthermore, applying linear mixed-effects models with correction for age, sex, and a random factor for subject ID also yielded no significant difference (Table S4). Comparing the tau load of αSyn + vs. αSyn- cases in 28 brain regions separately under correction of age and sex, there was no significant difference (Table S6). These findings support a theory of tau accumulation that is independent from α-syn deposits.

To examine whether tau distribution varies between α-syn-positive subgroups, we performed multiple linear regression controlling for specific region names, age, and sex (Fig. 3, Tables S9 and S11). After FDR correction, there was a significantly decreased tau load in αSyn + B compared to αSyn− cases in cortical (*p* < 0.001), hippocampal (*p* < 0.001), and amygdala–entorhinal regions (*p* = 0.021). Furthermore, but only with correction for age and sex, there was a significantly higher tau load in αSyn + A than in αSyn− cases across cortical regions (*p* = 0.004), indicating a positive association between α-syn in the amygdala and cortical tau accumulation. On the other hand, there was a significantly lower tau load in αSyn + C than in αSyn− cases across cortical regions (*p* = 0.022), which was also only evident when controlling for age and sex, suggesting a relatively lower cortical tau load at death when cortical α-syn load is apparent. These findings were comparable with additional statistical correction for ApoE4 carriage, except for the lower tau load of αSyn + B in the amygdala–entorhinal region.

### Aβ load in relation to α-syn distribution

**Fig. 4:**
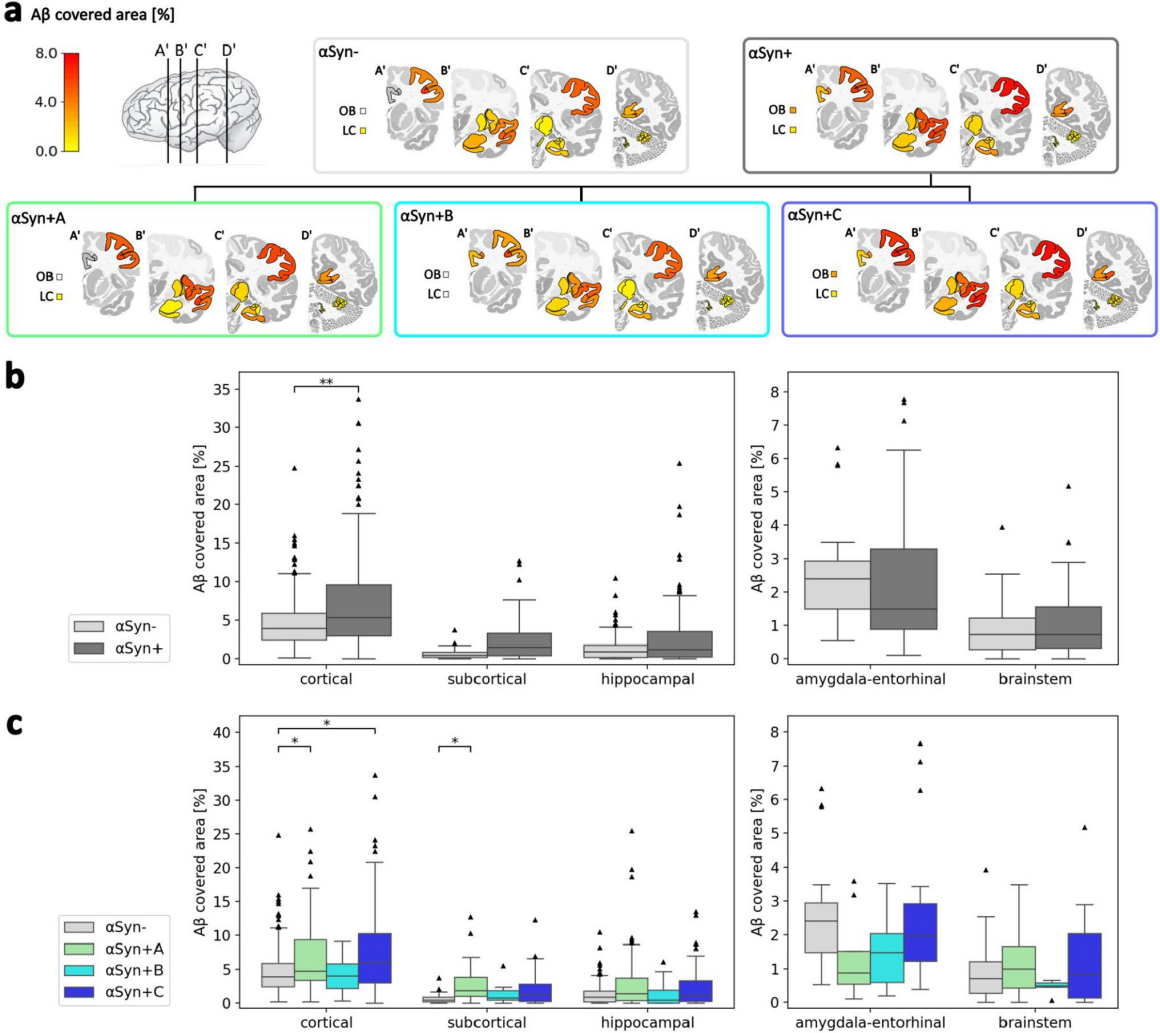
Amyloid beta (Aβ) load and distribution in Alzheimer’s disease. **a** Median Aβ-covered area of each α-syn distribution group and subgroup. **b** Comparison of the Aβ-covered area between αSyn− and αSyn + groups. **c** Comparison of the Aβ-covered area between αSyn− and αSyn + A (amygdala predominant), αSyn + B (brainstem predominant), and αSyn + C (cortical) α-syn-positive subgroups. Statistics in **b** and **c** were calculated with multiple linear regression across region clusters, controlling for region names, age, and sex, and false discovery rate correction. Boxplots complemented with scatter dots for female and male patients are available in the supplementary Fig. S8. *LC* locus coeruleus, *OB* olfactory bulb

The analyzed AD cases showed marked Aβ pathology, predominantly corresponding to Thal phase 5 (Table 1). The most affected areas were the parietal, frontal, and temporal cortices, followed by the amygdala, hippocampal, subcortical, and brainstem areas, which were impacted to a markedly lesser extent (Fig. 4, Table 5; refer to Table S7 for results per region). To examine potential associations between Aβ and α-syn loads, we compared the Aβ-covered area of αSyn− vs. αSyn + cases with multiple linear regression, correcting for the specific region name, age, and sex. There was a significantly higher Aβ load in cortical brain regions of αSyn + cases (Fig. 4, Table 5), suggesting an association of cortical Aβ with α-syn load.

**Table 5:** Comparison of the Aβ-covered area between αSyn + vs. αSyn− groups with multiple linear regression controlling for age and sex and correction for false discovery rate

Region cluster	n (αSyn−)	n (αSyn +)	Median [IQR] [%] of αSyn- cases	Median [IQR] [%] of αSyn + cases	β, *p*-value (age, sex corrected)
Cortical	149	288	**3.9** [2.2; 6]	**5.4** [3.0; 9.7]	*β* = 0.017, *p* = **0.003**
Subcortical	20	93	0.5 [0.2; 0.9]	1.5 [0.4; 3.3]	*β* = 0.012, *p* = 0.09
Hippocampal	98	226	0.9 [0.2; 1.8]	1.2 [0.3; 3.5]	*β* = 0.007, *p* = 0.09
Amygdala–entorhinal	16	31	2.4 [1.5; 2.9]	1.5 [0.9; 3.3]	*β* = − 0.002, *p* = 0.86
Brainstem	26	32	0.7 [0.3; 1.3]	0.7 [0.3; 1.6]	*β* < − 0.001, *p* = 0.86

Significant p-values and the highest median Aβ-covered area of αSyn− and αSyn + groups were labeled in bold

*ID* subject ID, *IQR* interquartile range

As control analyses, we conducted multiple linear regression without correction for sex and age. Again, there was a significant difference regarding the Aβ load between αSyn− and αSyn + groups in cortical regions (Table S4). Additionally, there were significantly higher Aβ covered areas in the subcortical and hippocampal regions, suggesting a positive association between Aβ and α-syn across regions. Supplementing the multiple linear regression model with ApoE4 next to sex, age, and region name, the cortical Aβ load showed a trend but was not significantly different (*p* = 0.077). In a further control analysis, applying linear mixed-effects models with correction for age, sex, and a random factor for subject ID, there was also no significant difference, probably due to overcorrection (Table S4). Regarding the 28 brain regions separately, the Aβ load was higher in the αSyn + vs. αSyn− group in the occipital sulcus, the insula cortex, and the parahippocampal gyrus; however, these effects did not remain significant after FDR correction or after correction for age and sex (Table S7). Thus, the increase of the Aβ load in αSyn + AD cases becomes particularly apparent when multiple regions are considered in one analysis; it is mostly evident in cortical areas, and the effect is partly explained by ApoE4 carriage.

To examine whether the increased Aβ load can be attributed to specific α-syn-positive subgroups, we applied multiple linear regression controlling for region names, age, and sex (Fig. 4, Table S11). After FDR correction, there was a significantly increased Aβ load in αSyn + A compared to αSyn− cases across cortical regions (*p* = 0.037) and subcortical regions (*p* = 0.048). Additionally, there was a significantly increased Aβ load in αSyn + C compared to αSyn- (*p* = 0.01) across cortical regions, suggesting that the finding described above of more cortical Aβ in αSyn + cases is mainly driven by α-syn subgroups αSyn + A and αSyn + C. With additional correction for ApoE4 carriage, there was a significantly higher Aβ load in the cortical regions of the αSyn + A vs. αSyn− (*p* = 0.023) and αSyn + B (*p* = 0.0024) and in the hippocampal region of αSyn + A vs. αSyn + C (*p* = 0.010), supporting the notion of a particularly higher Aβ load in αSyn + A.

Regarding the theory that depositional patterns can spread further, we plotted the values of mean cortical Aβ load and α-syn load in the amygdala against each other (Fig. S12). Subgroup αSyn + C appears to encompass subgroups αSyn + A and αSyn + B, making a development from αSyn + A and αSyn + B to αSyn + C conceivable.

### α-syn co-pathology in relation to age, sex, and ApoE genotype

**Fig. 5:**
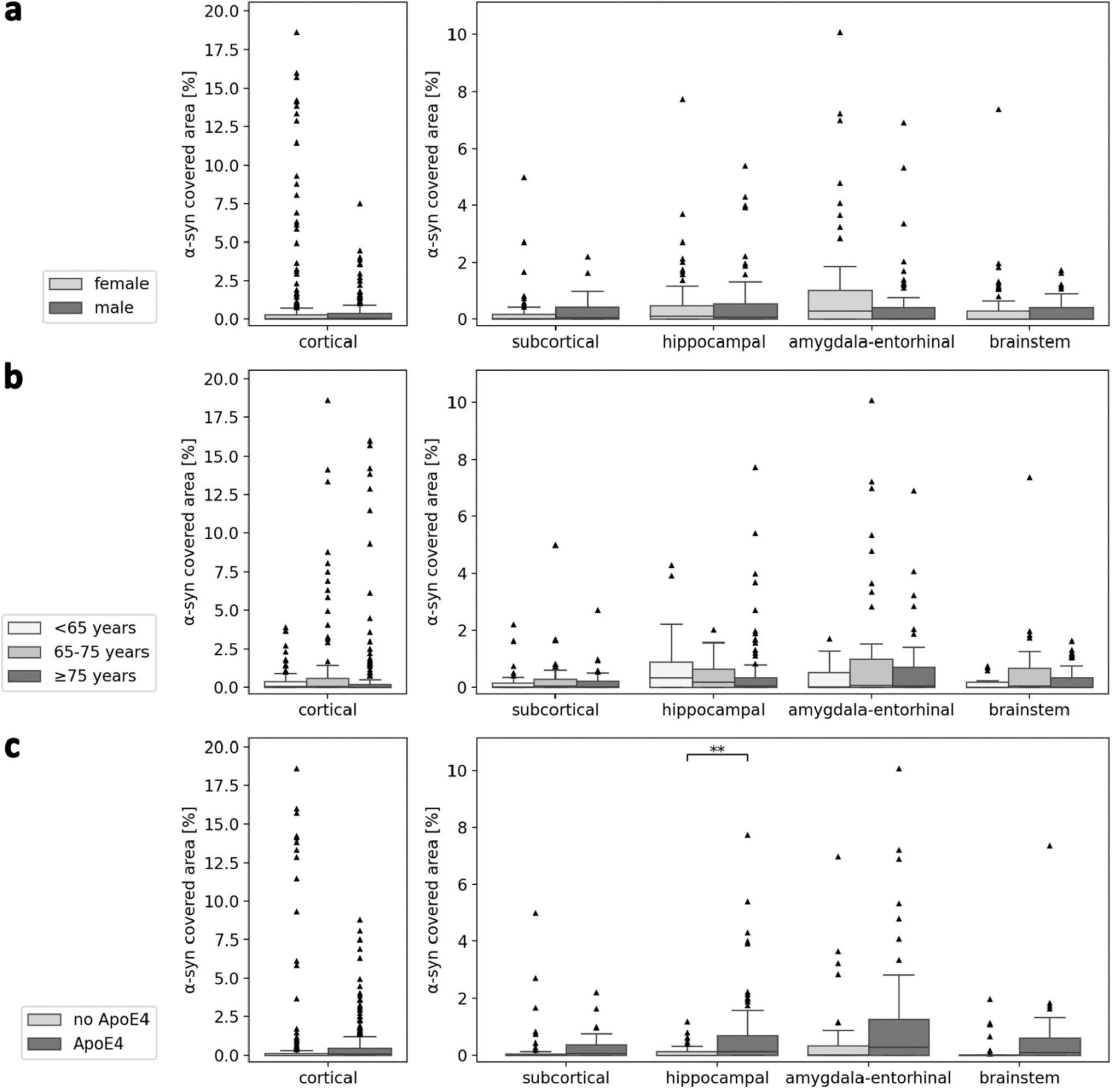
α-syn load split up by **a** sex, **b** age at death, and **c** ApoE genotype in Alzheimer’s disease cases. Statistics were calculated with multiple linear regression across region clusters, correcting for specific region names and false discovery rate correction. Results with age or sex correction are presented in the main text. Boxplots complemented with scatter dots for female and male patients are available in the supplementary Fig. S9. ApoE4 means that at least one ApoE4 allele is apparent

We examined the association of α-syn co-pathology in AD with age at death, sex, and ApoE status (Fig. 5). Although there is a recruitment bias in the cohort, comparing features within this cohort might provide further insight. In detail, we applied multiple linear regression with α-syn-covered area as the target variable and sex as a predictor variable across region clusters, controlling for the specific region names. An increased α-syn load in cortical regions in female vs. male cases (*β* = − 0.0049, *p* = 0.038) did not remain significant after FDR correction (*p* = 0.19) with comparable results after additionally correcting for age. Cortical, region-wise comparison within the αSyn + C group suggests that this finding is mainly outlier driven in the female group (Fig. S5) and a larger cohort would be needed for further clarification. The α-syn load also did not differ between female and male cases in other brain regions.

To examine the association with age, we defined three age groups: < 65 years at death (< 65), 65 to < 75 years (65–75), 75 years or older (≥ 75). Thereby, it should be noted that all cases pertain to advanced stages of AD. We applied multiple linear regression with α-syn-covered area as the target variable and age group as a predictor variable across region clusters, controlling for specific region names. Before FDR correction, there was a significantly higher cortical α-syn load in AD patients 65–75 years old compared with those  < 65 years (*β* = 0.007, *p* = 0.033). This result was not significant after FDR correction or correction for sex. Interestingly, there was a significantly lower α-syn load in the hippocampal region in AD patients 65–75 years old compared with those  < 65 years (*β* = − 0.0033, *p* = 0.030), which was also significant after correction for sex but not after FDR correction. The amygdala–entorhinal α-syn load was significantly lower in patients  ≥ 75 years old compared with those 65–75 years (*β* = − 0.0078, *p* = 0.033), which was also significant after correction for sex but not after FDR correction. In total, these results suggest that α-syn co-pathology in general appears independent of patient age, but a higher hippocampal and amygdala–entorhinal α-syn load might be associated with a younger age at death to a certain extent. Another explanation could be that younger patients with initiated protein deposition cascades can accumulate higher α-syn loads in the hippocampus and amygdala until death, maybe due to fewer life-limiting comorbidities. However, this trend was not reflected in cortical regions.

To evaluate the association of α-syn load in AD with the ApoE genotype, we compared AD cases with at least one ApoE4 allele to cases without ApoE4. Again, multiple linear regression was applied with α-syn load as the target variable and ApoE status as the predictor variable across region clusters, controlling for the specific region names. The cortical α-syn load of ApoE4 carriers was significantly lower (*β* = − 0.0055, *p* = 0.034), but did not remain significant after FDR correction or correction for sex and age. On the other hand, there was a significantly higher α-syn load in the hippocampal (*β* = 0.0045, *p* = 0.0019) and amygdala–entorhinal regions (*β* = 0.0066, *p* = 0.038) of ApoE4 carriers, which was significant after correction for age and sex, but only the difference in the hippocampal regions stayed significant after FDR correction (*p* = 0.009 without and *p* = 0.016 with correction for age and sex). These results suggest ApoE4 as a risk factor for higher hippocampal and putatively amygdala–entorhinal α-syn load, which in turn might be associated with a younger age at death.

### Aβ and tau load in relation to age, sex, and ApoE genotype

Additionally, we investigated the relation of age, sex, and ApoE genotype regarding tau and Aβ load (Figs. S10 and S11). In parallel to the α-syn analysis, the cohort selection is biased by voluntary donation, but an examination within the cohort might still be insightful. We applied multiple linear regression with tau or Aβ-covered area as the target variable and sex, age, or ApoE as predictor variables across region clusters, controlling for the specific region names and FDR correction. There was a significantly higher tau load in male patients in the hippocampal (*β* = 0.029, *p* = 0.009) and amygdala–entorhinal regions (*β* = 0.06, *p* = 0.009), which was also significant after correction for age (*p* = 0.022, respectively). Conversely, the Aβ load was significantly higher in female patients in cortical (*β* = −0.022, *p* < 0.001) and hippocampal regions (*β* = −0.013, *p* < 0.001), which was significant after correction for age. These findings suggest a sex imbalance toward tau in male and Aβ in female cases.

Regarding different age groups, all with advanced disease stages, there was a significantly lower cortical tau load in the oldest group (≥ 75 years at death) than in the younger age groups, < 65 (*β* = −0.024, *p* < 0.001) and 65–75 (*β* = −0.024, *p* = 0.038). Both findings were significant after correction for sex. In line with this observation, there was a significantly higher Aβ load in the youngest age group, < 65 years, than in 65–75 years old patients in the hippocampal regions (*β* = −0.016, *p* < 0.001) and in brainstem regions in the 65–75 (*β* = −0.015, *p* = 0.007) and ≥ 75 years cases (*β* = −0.007, *p* = 0.0027). The findings remained significant after correction for sex and suggest a higher deposit load in younger AD cases at death.

Concerning the presence of at least one ApoE4 allele, there was no significant association with tau covered areas, but with further age and sex correction, there was a significantly decreased tau load in ApoE4 carriers in cortical (*β* = −0.020, *p* = 0.03), hippocampal (*β* = −0.026, *p* = 0.03), and amygdala–entorhinal regions (*β* = −0.043, *p* = 0.049). Regarding Aβ, there was a higher Aβ load in the cortical regions of ApoE4 carriers (*β* = 0.019, *p* = 0.001), also significant after age and sex correction. This finding coincides with the high Aβ load in the αSyn + C cases with a relatively high proportion of ApoE4 carriers. ApoE4 might be related to disseminated α-syn deposition and to a higher cortical Aβ load with a speculative causal relationship.

## Discussion

Quantifying Aβ, tau, and α-syn load across brain regions in 72 Alzheimer’s disease (AD) patients, 60% of the cases showed detectable Lewy pathology. While the exact ratios vary between cohorts due to selection and recruitment biases [4, 30, 76], the main findings and descriptions of frequently pronounced co-pathologies are reinforced across studies. The α-syn deposit load predominates in the amygdala, but is heterogeneous in the cortical and brainstem regions, which can be subgrouped into several distribution patterns. The extent of Aβ and tau load varies between these α-syn subgroups, suggesting direct and indirect protein interactions and confounding factors.

### Alpha-synuclein distribution patterns: separate groups in a progressive process

Approaching previously specified Lewy body pathology patterns [5, 51], we assigned α-syn-positive (αSyn +) AD cases to three subgroups by thresholding the regional α-syn-covered areas. The biggest subgroup, αSyn + C, showed disseminated α-syn pathology at least somewhere in the cortex and a high amount in the amygdala. The second largest subgroup, αSyn + A, exhibits an amygdala-predominant α-syn pattern without significant Lewy pathology in the cortex. Finally, few AD cases mainly had α-syn deposits in the brainstem, αSyn + B, more specifically in the substantia nigra and to a lesser extent in the locus coeruleus. This classification approximates previously described amygdala-predominant and disseminated α-syn distribution patterns in AD [76].

While αSyn + A presents with Lewy pathology in the amygdala but not in the brainstem, αSyn + B shows higher loads in the brainstem than in the amygdala. Thus, αSyn + A and αSyn + B seem to be separated from each other. The prevalence of αSyn + B was relatively low in the study cohort, but might still be artificially increased due to voluntary brain donation. As the case collection is not population representative, it remains speculative if αSyn + B equals a random coexistence of AD and Parkinson’s disease, with a prevalence of Parkinson’s disease of around 1.6% in Europe between 70 and 79 years [59].

Focusing on the α-syn loads across regions, αSyn + A and αSyn + B can theoretically develop into the pattern of αSyn + C, supporting the theory of spreading Lewy pathology as far as these conclusions are possible from non-longitudinal autopsy data [1, 10]. This theory might be further supported by the finding of more αSyn + C cases in comparison with αSyn + A or αSyn + B patterns in genetic cases and cases with young age at symptom onset. Patients with AD-related mutations frequently showed an αSyn− pattern (47%) or a disseminated co-pathology, αSyn + C (37%), but much less common intermediate αSyn + A (11%) and αSyn + B (5%) states. Although numbers are small, this distribution supports the hypothesis that strong genetic Aβ drive may accelerate or alter the trajectory of α-synuclein propagation. In this framework, AD-related mutations could hasten the transition from early phases (αSyn + A/αSyn + B) into widespread cortical α-synuclein deposition (αSyn + C), within the same disease duration. Conversely, some mutation carriers remain αSyn− despite dominant Aβ pathology. Thus, Aβ- and α-synuclein-driven processes may arise separately but converge in a subset of patients, shaping overall disease tempo and severity.

Lacking olfactory bulb tissue in a high number of cases did not allow for detection of rare cases with olfactory only Lewy pathology described by Attems et al. [5]. Additionally, a bigger cohort would be needed to detect a limbic-predominant subgroup, which is probably currently included as part of the cortical subgroup.

It is noticeable that the amygdala was the most affected region by α-syn deposits in AD, followed by the CA2 region of the hippocampus. This finding is apparent across subgroups except for some brainstem-predominant cases. While the amygdala's predominance of α-syn in AD was described before [4, 30, 76], the reasons for the region's sensitivity are still under discussion [56]. Nevertheless, distinct Lewy pathology distributions described in DLB [51, 52] are also present in AD as a spectrum of co-pathology patterns related to partly overlapping clinical symptoms [18, 77].

### Tau load varies between α-syn subgroups

Comparing the (AT8-) hyperphosphorylated tau load of αSyn + vs. αSyn− AD cases with multiple linear regression correcting for age and sex, there was no significant difference. This finding is consistent with previous immunohistochemical analyses showing comparable tau loads in AD with and without Lewy body co-pathology [29]. However, comparing αSyn− cases with three α-syn-positive subgroups, there were significant differences, emphasizing the importance of patient stratification. We found a significantly increased cortical tau load in the amygdala-predominant α-syn subgroup, αSyn + A, compared to the αSyn- group, while the cortical tau load was lower in αSyn + B and αSyn + C. These results demonstrate a variable association between α-syn and tau load depending on the α-syn distribution and highlight the importance of statistical adjustment for age and sex.

Especially within the amygdala, some neurons contain Lewy bodies and neurofibrillary tangles concomitantly [43, 65]. A co-localization was also described in astrocytes [29]. Arai and colleagues argue that not all α-syn aggregations are Lewy bodies; on the other hand, the tau load might impact which regions develop more Lewy bodies [4]. The molecular relationship between α-syn and tau and its consequences is still under discussion, with several studies claiming adverse interactions between these proteins: α-syn and tau share molecular similarities and overlap in their radius of action [55]. Specific α-syn and tau isoforms show heightened binding affinities toward each other [27, 61]. α-syn plays a role in tau phosphorylation, and the proteins promote each other’s fibrillization [32, 36, 55]. There are further hints of α-syn driving tau accumulation through genetic elements responsible for higher baseline *SNCA* expression [69]. Ultimately, subjects with a positive cerebrospinal fluid α-syn seed aggregation assay had higher tau PET signals [28].

The increased cortical tau load in αSyn + A fits the hypothesis of mutual α-syn–tau interactions. The brainstem-predominant α-syn pattern seems to drop out of general patterns like amygdala predominance and therefore might correspond to separate mechanisms. The cortical α-syn subgroup showed a decreased tau load with a tendency for a younger age at death [58]. An explanation could be that AD with disseminated α-syn pathology is fatal before the tau load reaches levels as high as in α-syn-negative AD cases. α-syn may add to the toxic effect of hyperphosphorylated tau, so clinical relevance is already reached at a lower tau level.

### Aβ load is increased in α-syn subgroups

Comparing the Aβ load of αSyn + vs. αSyn− AD cases with multiple linear regression correcting for age and sex, there was an increased Aβ load in α-syn-positive cases attributable to αSyn + A and partly αSyn + C subgroups. There was also a trend of higher Aβ load in subcortical and hippocampal regions, supporting a general tendency. Such a positive association between Aβ and α-syn was partly described before in the clinical spectrum of AD and dementia with Lewy bodies [77] and the other way around in Lewy body dementia with Aβ co-pathology [54]. This finding also accords with semiquantitative studies revealing a strong association between AD pathology and amygdala-predominant α-syn deposition, while there was no such effect in a caudo-rostral α-syn co-pathology group [62]. On a more mechanistic level, several studies support an association between Aβ and α-syn [47]. Nuclear magnetic resonance spectroscopy suggests the interaction of Aβ with membrane-associated α-syn [46]. In vitro and in vivo experiments support the hypothesis that Aβ promotes α-syn aggregation [38, 48]. However, this hypothesis did not apply for specific regions; e.g., the Aβ load was not increased in the amygdala of the amygdala-predominant or cortical α-syn subgroups. These findings suggest a more complex interplay of Aβ and α-syn, as well as tau to a certain extent, involving multiple factors, rather than local correlations.

Recent studies align with this interpretation. Clinical and imaging studies demonstrated that α-syn co-pathology is more prevalent in advanced AD and accelerates amyloid-driven tau aggregation, thereby worsening clinical decline [2, 28]. Struebing et al. [69] further showed that AD cases with cortical α-synuclein carry higher Parkinson’s disease polygenic risk as well as increased AD age-at-onset risk, indicating contributions from both Parkinson’s disease (PD) and AD-related susceptibility factors. Together, these findings support a model in which Aβ, tau, and α-synuclein interact along partially distinct routes that converge in some patients, driving aggressive cortical disease and underscoring the importance of genetic and pathological stratification in future studies.

### Association of ApoE4, sex, and age with AD and α-syn co-pathology

Comparing epidemiological data in terms of age, sex, and ApoE genotype among α-syn groups and subgroups in AD, no significant differences were apparent. Although the study cohort is limited by voluntary recruitment, the finding is in line with previous observations [63]. However, regarding the ApoE genotype, there was a trend toward more ApoE4 carriers in the partition with α-syn deposits, more specifically in the αSyn + C cases. Comparing ApoE4 carriers with no ApoE4 carriers using multiple linear regression across regions, the ApoE4 allele was associated with a significantly higher α-syn load in the hippocampus. Additionally, the ApoE4 allele was associated with a higher cortical Aβ load and a lower tau load in several brain regions after age and sex correction. These results are supported by literature, presenting ApoE4 as a risk factor for AD [24, 44] with the specific effects on Aβ and tau differing between studies, approaches, and brain regions [6, 23, 31, 50, 68]. ApoE4 is also a risk factor for DLB [12] and increased α-syn levels in the cerebrospinal fluid of AD patients [75].

Regarding sex differences, there was a trend toward more female subjects in the partition with α-syn deposits, mostly apparent in the αSyn + A subgroup. Examining the association of the α-syn load in AD with sex across brain regions using multiple linear regression, there was no significant difference, suggesting that male and female patients show comparable α-syn load. This is in line with more or less sex-balanced cohorts in DLB [60]. Besides, we observed an increased Aβ load in female patients and an increased tau load in male patients predicted by multiple linear regression across brain regions. These findings align with the observations in a transgenic mouse model [34]. Several studies in humans found increased Aβ and tau load in women [8, 26, 57]. The discrepancy in the tau results may be attributable to differences in age distributions and analytical approaches.

The AD subgroup with disseminated cortical α-syn tended to have a lower mean age at death, consistent with previous observations [58]. Including all AD cases, the α-syn load did not differ significantly between age groups tested with multiple linear regression. This is in line with observations that α-syn co-pathology is common in sporadic, but also in younger genetic cases [43] and, thus, cannot be explained by simple accumulation with age. Comparing Aβ and tau load in AD between different ages with multiple linear regression across brain regions, the Aβ and tau load were focally increased in patients with a younger age at death. These results are partly in accordance with previous PET analyses which showed increased tau accumulation in younger Aβ-positive subjects, while Aβ deposition was faster in older cases [68]. In agreement with this finding, a PET study by Lowe et al. reported increasing tau load with age in cognitively unimpaired samples, but a higher tau load in younger cognitively impaired patients, suggesting higher loads in younger-onset AD [45]. In line with our results, there was also a higher Aβ load described in PETs of early-onset AD cases in comparison with late-onset cases [40].

In summary, subject to limited brain donation recruitment, ApoE4 is a risk factor for higher α-syn and Aβ load in AD. The exact causal chain remains speculation: ApoE4 might indirectly increase the α-syn load via increased Aβ and maybe tau loads [20] and directly via altered degradation of α-syn [53]. In particular, ApoE4 carriage statistically explained the association between αSyn + C and cortical Aβ load, but not the association between αSyn + A and cortical Aβ load, suggesting ApoE4 as a confounding factor and additional potentiating effects between deposit types. Aβ and tau load were partly increased in younger patients with dementia compared to older cases. This might be explained by the Aβ-related, genetically driven cases in our cohort, putatively together with higher body reserves in younger people. Alpha-synuclein co-pathology appears across all ages. The findings emphasize the importance of control for age and sex in research analyses, and especially in clinical diagnostics and therapies [25]. However, the results also show a pronounced complexity of the relationships. Despite subdivision into subgroups, there is often a large variance between the cases. Normative approaches and larger cohorts can help narrow down other factors that influence the extent of pathologies at an individual level.

### Strengths and limitations

A strength of this study is the large size of the dataset, comprising many brain regions analyzed in up to three immunohistochemical stains. The region annotation was standardized, and the deposit detection was automated to gain reliable and objective quantifications. The obtained segmentations can also serve as initial data for further morphology-based categorization of deposits. Despite this extensive approach, the study has several limitations. First, the dataset could be even larger and more complete regarding the availability of α-syn stains across brain regions to be more sensitive for smaller α-syn subgroups and to reduce the potential bias of missing stains. For example, a pure olfactory α-syn subgroup was not detected in our cohort, probably because of its rare appearance and incomplete tissue embedding in a subset of cases; additionally, the sex, age, and ApoE4 evaluation are limited by a relatively small and probably not representative cohort for epidemiological analyses. For precise proportions, population-based studies are necessary, and cross-ethnic datasets are needed. Second, the region annotation protocol focuses on small rectangles of regions of interest instead of whole slide images, which could miss variability within each block, e.g., neuronal degeneration is not homogeneous within the substantia nigra [21], which exceeds the approach of this article. However, this reduction helped to limit the amount of large-sized data and led to a reasonable consumption of computational power. Third, this study focused on specific antibody clones, namely clone 42 for α-syn, clone 4G8 for Aβ, and clone AT8 for tau staining. These antibodies are typically applied in diagnostics but are restricted to specific targets, e.g., AT8 sticks to tau with defined phosphorylation sites. Further studies are needed for other epitopes and to take other co-pathologies like TDP43 deposits into account. Finally, this analysis approached different AD neuropathological subgroups. However, the dataset exclusively represents advanced stages of AD, and the α-syn subgrouping did not fully explain the heterogeneity in tau and Aβ load. As a correlative post-mortem study, causal conclusions remain speculative.

## Conclusion

Quantifying neuropathological deposits in Alzheimer’s disease, we found α-syn co-pathology in more than half of the cases across age groups with a tendency toward female patients and an association with the ApoE4 allele. Assigning three distinct α-syn distribution groups, the common amygdala-predominant and cortical α-syn patterns were associated with an increased cortical Aβ load, while tau load varied between these groups. To conclude, next to age, sex, and ApoE, the α-syn distribution pattern is associated with distinct Aβ and tau loads with potential therapeutic relevance in immunization therapies.

## Supplementary Information

Below is the link to the electronic supplementary material.

Supplementary file1 (PDF 2155 KB)

## Data Availability

Supporting data, code, and segmentation models applied in this study can be obtained from the corresponding author upon reasonable request. The code to plot colors on brain atlas images is publicly available on GitHub (https://github.com/cor2ni/2D_brain_plot).

## Abbreviations

α-syn: Alpha-synuclein
αSyn-: Alpha-synuclein deposit negative
αSyn +: Alpha-synuclein deposit positive
αSyn + A: Amygdala-predominant alpha-synuclein deposition
αSyn + B: Brainstem-predominant alpha-synuclein deposition
αSyn + C: Cortical alpha-synuclein deposition
Aβ: Amyloid beta
AD: Alzheimer’s disease
DLB: Dementia with Lewy bodies
FDR: False discovery rate
IQR: Interquartile range
PD: Parkinson’s disease
