# Supplementary material for: Alpha-synuclein deposition patterns in Alzheimer’s disease: association with cortical amyloid beta and variable tau load
Source: Acta Neuropathol. 2025 Oct 30;150(1):46. doi: 10.1007/s00401-025-02952-w (PMC12575528; doi:10.1007/s00401-025-02952-w)
Supplement: Supplementary file 1 — Supplementary file1 (PDF 2155 KB) [file 401_2025_2952_MOESM1_ESM.pdf]

Supplement for

Title:

Alpha-synuclein deposition patterns in Alzheimer's disease: association with cortical amyloid beta and variable tau load

Authors:

Antonia Neubauer[1,2], Doris Weissenbrunner [1,2], Susanna Pekrun [1,2], Sigrun Roeber[1,2], Viktoria Ruf [1,2], Paul Feyen[1,2], Felix L. Strübing[1,2], Jochen Herms[1,2,3]

Affiliations:
[1]Center for Neuropathology and Prion Research, Ludwig Maximilians University of Munich, Munich, Germany
[2]German Center for Neurodegenerative Diseases (DZNE), Munich, Germany
[3]Munich Cluster for Systems Neurology (SyNergy), Munich, Germany

Corresponding author:

Antonia.Neubauer@med.uni-muenchen.de

Table of Contents

Supplementary Tables

Table S1 Overview of 28 annotated brain regions. 3
Table S2 Overview of the primary antibodies applied in this study. 4
Table S3 Overview of genetic and sporadic Alzheimer's disease (AD) cases. 4
Table S4 Comparison of the α-syn, tau, and Aβ covered area between αSyn- vs. αSyn+ groups. 4
Table S5 Region-wise comparison of the α-syn covered area between αSyn- vs. αSyn+ groups. 5
Table S6 Region-wise comparison of the tau covered area between αSyn- vs. αSyn+ groups. 6
Table S7 Region-wise comparison of the Aβ covered area between αSyn- vs. αSyn+ groups. 7
Table S8 Available n, median and interquartile range of α-syn covered area of α-syn subgroups 7
Table S9 Available n, median and interquartile range of tau covered area of α-syn subgroups 8
Table S10 Available n, median and interquartile range of Aβ covered area of α-syn subgroups 8
Table S11 Comparison of αSyn- cases (-) vs. α-syn positive subgroups. 8

Supplementary Figures

Fig. S1 Deposit quantification in the substantia nigra (SN) and locus coeruleus (LC). 10
Fig. S2 Accuracy of the ilastik random forest pixel classifiers tested with ten test images. 11
Fig. S3 Age at clinically reported symptom onset, disease duration, and age at death of Alzheimer's disease patients with and without Lewy body co-pathology. 12
Fig. S4 Clinical diagnoses of patients with neuropathologically confirmed extensive Alzheimer's disease related neuropathological change. 12
Fig. S5 Boxplots comparing cortical α-synuclein (α-syn) deposit loads in female vs. male Alzheimer's disease (AD) patients with cortical Lewy pathology (αSyn+C). 13
Fig. S6 Alpha-synuclein (α-syn) load and distribution in Alzheimer's disease cases. 14
Fig. S7 Tau load and distribution in Alzheimer's disease. 15
Fig. S8 Amyloid beta (Aβ) load and distribution in Alzheimer's disease. 16
Fig. S9 α-Syn load split up by (a) sex, (b) age at death, and (c) ApoE genotype in Alzheimer's disease cases. 17
Fig. S10 Tau load split up by (a) sex, (b) age at death and (c) ApoE genotype in Alzheimer's disease cases. 18
Fig. S11 Aβ load split up by (a) sex, (b) age at death and (c) ApoE genotype in Alzheimer's disease cases. 19
Fig. S12 Overview of cortical Aβ load vs. α-syn load in the amygdala. 20

Supplementary Tables

Table S1 *Overview of 28 annotated brain regions.*

*Regions are listed with size and annotation comments as well as the attribution to a region cluster. Representative areas without artifacts or large blood vessels were selected for all regions. For cortical regions, the annotation is aligned vertically to the brain surface to cover all cortical layers. "Number" refers to the numbering in Fig. 1 of the main manuscript. One "tile" equals 4096*4096 pixels (900 x 900 µm²). Cerebellar regions and the olfactory bulb were not included in region clusters due to the limited number of available stainings in these regions.*

Number	Region	Comment	Label size (approximately)	Region cluster
1	middle frontal gyrus	at the level of the nucleus accumbens	3x0.5 tiles	cortical
2	sulcus of middle frontal gyrus		3x0.5 tiles	cortical
3	cingulate gyrus, rostral (anterior) part		3x0.5 tiles	cortical
4	sulcus between cingulate and superior frontal gyrus	on the side of frontal gyrus	3x0.5 tiles	cortical
5	long and short insular gyri	at the level of basal ganglia with putamen, globus pallidus and corpus of the caudate nucleus	3x0.5 tiles	cortical
6	edge between ventral and dorsal claustrum		1x1 tile	subcortical
7	putamen		1x1 tile	subcortical
8	superior temporal gyrus	at the level of the amygdala	3x0.5 tiles	cortical
9	sulcus between superior and middle temporal gyrus on the side of the middle gyrus		3x0.5 tiles	cortical
10	middle temporal gyrus		3x0.5 tiles	cortical
11	amygdala (lateral nucleus)	at the level of (trans-)entorhinal cortex	1x1 tile	amygdala-entorhinal
12	entorhinal cortex		3x0.5 tiles	amygdala-entorhinal
13	parietal gyrus	inferior parietal lobulus at the level of the splenium	3x0.5 tiles	cortical
14	mediodorsal nucleus of thalamus	at the level of substantia nigra	1x1 tile	subcortical
15	lateral nucleus of thalamus		1x1 tile	subcortical
16	substantia nigra, pars compacta	representative areas avoiding pigmented neurons	2 x 0.25 tiles	brainstem
17	CA4 region of hippocampus	at the level of the lateral geniculate nucleus	1x1 tile	hippocampal
18	CA3 region of hippocampus		1x1 tile	hippocampal
19	CA2 region of hippocampus		1x1 tile	hippocampal
20	CA1 region of hippocampus		1x1 tile	hippocampal
21	subiculum		1x1 tile	hippocampal
22	parahippocampal gyrus		3x0.5 tiles	hippocampal
23	striate area gyrus	Broca areal 17	3x0.5 tiles	cortical
24	striate area sulcus		3x0.5 tiles	cortical
25	cerebellar cortex	at the level of the dentate nucleus	1x1 tile	
26	cerebellum, dentate nucleus		1x1 tile	
LC	locus coeruleus	representative areas avoiding pigmented neurons	2 x 0.25 tiles	brainstem
OB	olfactory bulb		1x1 tile	

LC locus coeruleus, *OB* olfactory bulb

Table S2 *Overview of the primary antibodies applied in this study.*

Antibody	Clone	Company	Catalog #	Dilution
α-Synuclein	42	BDTransduction	610787	1:1000
Phospho-Tau (Ser202, Thr205)	AT8	ThermoFisher	MN1020	1:400
β-Amyloid	4G8	BioLegend	800701	1:5000

Table S3 *Overview of genetic and sporadic Alzheimer's disease (AD) cases.*

Detected variants and ratios are listed for αSyn- and αSyn+ groups and for α-synuclein distribution subgroups. The effect of ApoE4 carriage is analyzed separately and not included in this table.

	**All**	**αSyn-**	**αSyn+**	**αSyn+A**	**αSyn+B**	**αSyn+C**
n with genetic screening / total n	69 / 72	27 / 29	42 / 43	14 / 15	5 / 5	22 / 22
APP<br>PSEN1<br>PSEN2<br>TREM2	3<br>13<br>2<br>1	2<br>5[a]<br>1<br>1	1<br>8<br>1<br>-	-<br>1<br>1<br>-	-<br>1<br>-<br>-	1<br>6<br>-<br>-
**Sum (genetic AD)**<br>% of genetic AD cases	19<br>100 %	9<br>47 %	10<br>53 %	2<br>11 %	1<br>5 %	7<br>37 %
**Sum (sporadic AD)[b]**<br>% of sporadic AD cases	53<br>100 %	20<br>38 %	33<br>62 %	13<br>25 %	4<br>8 %	15<br>28 %

[a]In one patient a TREM2 mutation was found in addition to a PSEN1 mutation. This patient is listed here only with the PSEN1 mutation.

[b]In this overview, "sporadic" AD includes cases with genetic screening without detection of a mutation as well as cases that had no high suspicion of a mutation, e.g., due to late-onset dementia, and did not receive genetic screening.

Comparing the α-synuclein positivity between genetic and sporadic AD cases, there was no significant difference according to a chi-squared test (chi-square statistic = 0.54, p = 0.46). Regarding the αSyn+ subgroups, the amygdala predominant pattern, αSyn+A, seems to be more common in sporadic AD cases. However, comparing the distribution of sporadic and genetic AD cases across α-synuclein distribution groups (αSyn-, αSyn+A, αSyn+B, αSyn+C) with a chi-squared test, there was also no significant difference (chi-square statistic = 2.05, p = 0.56), suggesting comparable Lewy co-pathology distributions.

Table S4 *Comparison of the α-syn, tau, and Aβ covered area between αSyn- vs. αSyn+ groups.*

Groups were compared with multiple linear regression without correction for age, sex, and subject ID, and with linear mixed effects models with correction for these factors. Effect size (β) and p-value are presented, respectively.

**Region cluster**	**α-syn β, p (uncor.)**	**α-syn β, p (age, sex, ID cor.)**	**tau β, p (uncor.)**	**tau β, p (age, sex, ID cor.)**	**Aβ β, p (uncor.)**	**Aβ β, p (age, sex, ID cor.)**
cortical	β=0.010, **p=0.003**	β=0.009, p=0.15	β=-0.009, p=0.46	β=-0.016, p=0.68	β=0.023, **p<0.001**	β=0.014, p=0.33
subcortical	β=0.003, **p=0.025**	β=0.003, p=0.07	β=0.003, p=0.68	β=-0.003, p=0.69	β=0.015, **p=0.022**	β=0.012, p=0.33

hippocampal	β=0.005, **p=0.003**	β=0.005, p=0.07	β=-0.017, p=0.22	β=-0.015, p=0.68	β=0.010, **p=0.016**	β=0.007, p=0.48
Amygdala-entorhinal	β=0.014, **p<0.001**	β=0.013, **p<0.001**	β=-0.046, p=0.11	β=-0.044, p=0.29	β=-0.003, p=0.69	β=-0.003, p=0.85
brainstem	β=0.005, **p=0.003**	β=0.005, p=0.07	β=-0.003, p=0.64	β=-0.002, p=0.69	β=0.002, p=0.69	β=-0.0005, p=0.85

All p-values were FDR corrected. Significant p-values were labeled in bold. *cor.* corrected, *FDR* false discovery rate, *ID* subject ID, *p* p-value, *uncor.* Uncorrected

Table S5 *Region-wise comparison of the α-syn covered area between αSyn- vs. αSyn+ groups.*

Groups were compared with multiple linear regression controlling and not controlling for age and sex.

Region name	n (αSyn-)	n (αSyn+)	Median [IQR] [%] of αSyn- cases	Median [IQR] [%] of αSyn+ cases	p (uncor.)	p (age, sex cor.)
medial frontal gyrus	8	39	0.001 [0.0005; 0.0019]	0.034 [0.0058; 0.15]	0.5	0.53
frontal sulcus	8	38	0.001 [0.0004; 0.0017]	0.039 [0.0049; 0.27]	0.47	0.47
cingulate gyrus	8	34	0.0021 [0.0002; 0.0048]	0.23 [0.0515; 1.34]	0.35	0.32
cingulate sulcus	8	35	0.0009 [0.0006; 0.0024]	0.23 [0.0113; 1.26]	0.35	0.32
parietal gyrus	7	39	0.0013 [0.0005; 0.0022]	0.0596 [0.0028; 0.28]	0.35	0.32
superior temporal gyrus	9	38	0.0007 [0.0004; 0.0024]	0.19 [0.012; 0.53]	0.4	0.38
temporal sulcus	9	37	0.001 [0.0005; 0.0068]	0.19 [0.023; 0.73]	0.39	0.37
medial temporal gyrus	9	35	0.0021 [0.0003; 0.0037]	0.102 [0.015; 0.46]	0.38	0.35
occipital gyrus	1	10	0.006 [0.006; 0.006]	0.0043 [0.0012; 0.055]	0.66	0.32
occipital sulcus	1	10	0.0032 [0.0032; 0.0032]	0.0037 [0.002; 0.096]	0.66	0.61
insula cortex	5	33	0.0005 [0.0004; 0.0057]	0.4092 [0.027; 1.35]	0.35	0.32
claustrum	9	36	0.0002 [0.0001; 0.0014]	0.16 [0.017; 0.42]	0.35	0.32
putamen	9	38	0.0037 [0.0001; 0.0086]	0.059 [0.016; 0.49]	0.35	0.32
hippocampus CA1	5	30	0.0004 [0.0001; 0.0008]	0.15 [0.012; 0.48]	0.37	0.32
hippocampus CA2	5	29	0.0001 [0.0001; 0.0041]	**0.58** [0.094; 1.32]	0.35	0.32
hippocampus CA3	5	28	0.0003 [0.0003; 0.001]	0.10 [0.017; 0.35]	0.35	0.32
hippocampus CA4	5	30	0.0008 [0.0004; 0.0011]	0.046 [0.0048; 0.31]	0.35	0.32
subiculum	5	30	0.0008 [0.0005; 0.0066]	0.16 [0.038; 0.78]	0.35	0.32
parahippocampal gyrus	5	29	0.0011 [0.0004; 0.0038]	0.24 [0.022; 0.79]	0.35	0.32
thalamus medial nucl.	2	12	0.0005 [0.0002; 0.0007]	0.026 [0.012; 0.13]	0.4	0.32
thalamus ventrolat. nucl.	2	11	0.0007 [0.0004; 0.0011]	0.024 [0.0022; 0.11]	0.47	0.32
cerebellar cortex	0	7	nan [nan; nan]	0.0011 [0.0002; 0.0023]	nan	nan
cerebellum dentate nucl.	0	7	nan [nan; nan]	0.006 [0.0024; 0.0065]	nan	nan
substantia nigra	17	33	0.0047 [0.0008; 0.0064]	0.37 [0.027; 1.1]	**0.004**	**0.005**
locus coeruleus	19	39	0.0014 [0.0005; 0.0044]	0.11 [0.014; 0.36]	0.35	0.32
amygdala	27	37	0.0006 [0.0001; 0.0117]	**1.01** [0.415; 2.04]	**0.004**	**0.005**
entorhinal gyrus	27	33	0.0019 [0.0008; 0.0052]	0.46 [0.14; 1.16]	**0.013**	**0.023**
olfactory bulb	9	15	0.0009 [0.0003; 0.0272]	**0.51** [0.32; 0.89]	**0.014**	**0.023**

All p-values were FDR corrected. Significant p-values and the three highest median α-syn loads of the αSyn+ group were labeled in bold. *cor.* corrected, *FDR* false discovery rate, *IQR* interquartile range, *nan* not enough values available, *p* p-value, *uncor.* Uncorrected

Table S6 *Region-wise comparison of the tau covered area between αSyn- vs. αSyn+ groups.*

Groups were compared with multiple linear regression controlling and not controlling for age and sex.

Region name	n (αSyn-)	n (αSyn+)	Median [IQR] [%] of αSyn- cases	Median [IQR] [%] of αSyn+ cases	p (uncor.)	p (age, sex cor.)
medial frontal gyrus	22	34	18.8 [12.4; 23]	15.5 [6.2; 22.6]	0.88	0.9
frontal sulcus	23	31	21.5 [11.7; 25.5]	15.1 [4.8; 23.9]	0.88	0.77
cingulate gyrus	12	28	13.7 [6; 20.5]	15.4 [5.5; 25.4]	0.88	0.98
cingulate sulcus	12	27	12 [8.1; 21.7]	13.1 [6.2; 22.1]	0.97	0.9
parietal gyrus	8	15	11.3 [7.6; 15.9]	**22.5** [11.7; 28.4]	0.72	0.77
superior temporal gyrus	28	42	17.4 [11.4; 23.3]	14.4 [9.5; 22.3]	0.88	0.9
temporal sulcus	27	41	21.3 [16.1; 26.9]	16.8 [11.4; 24.5]	0.72	0.77
medial temporal gyrus	28	39	18.7 [14.1; 25.3]	17.7 [10.1; 25.7]	0.88	0.9
occipital gyrus	25	41	4.6 [1.8; 7.8]	3.9 [0.5; 7.8]	0.88	0.9
occipital sulcus	25	41	6.9 [2.7; 10.2]	4.9 [1.3; 11.9]	0.93	0.98
insula cortex	6	16	15.9 [12.1; 22.3]	**22.3** [11.4; 24.9]	0.88	0.9
claustrum	8	19	7.3 [5.8; 9.8]	6.6 [3.1; 12.6]	0.97	0.9
putamen	9	19	2.5 [1.5; 4.2]	1.8 [0.5; 4.2]	0.97	0.9
hippocampus CA1	23	38	24.2 [16; 26.3]	19.6 [15.7; 27.6]	0.88	0.9
hippocampus CA2	21	36	25.9 [14.6; 30.3]	21.9 [14; 32.2]	0.88	0.9
hippocampus CA3	23	35	12.8 [6.2; 19.1]	16.2 [6.2; 21.3]	0.88	0.9
hippocampus CA4	22	38	13.3 [5.8; 19.8]	11.9 [5.9; 20.3]	0.97	0.98
subiculum	23	38	**32.1** [26; 36.4]	**26.3** [17.7; 35.4]	0.6	0.72
parahippocampal gyrus	21	37	18.2 [15.2; 21.2]	16.8 [10.1; 24]	0.91	0.98
thalamus medial nucl.	11	17	1.5 [0.6; 3.5]	1.6 [0.5; 3.5]	0.88	0.98
thalamus ventrolat. nucl.	11	17	0.5 [0.2; 1]	1 [0.3; 1.4]	0.88	0.9
cerebellar cortex	4	14	0 [0; 0]	0 [0; 0.1]	0.91	0.9
cerebellum dentate nucl.	4	14	0.1 [0; 0.1]	0 [0; 0.1]	0.88	0.9
substantia nigra	15	24	2.4 [0.018; 4.2]	1.6 [1.1; 2.5]	0.88	0.9
locus coeruleus	20	31	2.4 [0.017; 3.5]	2.2 [1.5; 3.1]	0.97	0.98
amygdala	21	33	**25.9** [0.188; 35.8]	19.8 [12.7; 31.7]	0.88	0.85
entorhinal gyrus	21	30	**26.8** [0.176; 32.7]	18.7 [11.5; 25.4]	0.65	0.77
olfactory bulb	10	14	5.6 [0.037; 10.3]	6.8 [3.5; 8.5]	0.97	0.98

All p-values were FDR corrected. The three highest median tau loads of αSyn+ and αSyn- groups were labeled in bold. *cor.* corrected, *FDR* false discovery rate, *IQR* interquartile range, *p* p-value, *uncor.* Uncorrected

Table S7 *Region-wise comparison of the Aβ covered area between αSyn- vs. αSyn+ groups.*

Groups were compared with multiple linear regression controlling and not controlling for age and sex.

Region name	n (αSyn-)	n (αSyn+)	Median [IQR] [%] of αSyn- cases	Median [IQR] [%] of αSyn+ cases	p (uncor.)	p (age, sex cor.)
medial frontal gyrus	20	37	3.6 [2.8; 5.8]	4.9 [2.9; 9.5]	0.36	0.57
frontal sulcus	21	34	**6.4** [3.9; 11.3]	**6.5** [4.6; 13.8]	0.65	0.99
cingulate gyrus	0	4	nan [nan; nan]	1.9 [1.6; 2.3]	nan	nan
cingulate sulcus	0	4	nan [nan; nan]	3.5 [2.3; 5.1]	nan	nan
parietal gyrus	15	26	**4.7** [3.4; 6.1]	**7.7** [4.4; 9.2]	0.39	0.57
superior temporal gyrus	14	28	3.7 [1.6; 5.8]	5.9 [3.4; 9]	0.36	0.57
temporal sulcus	13	27	5 [2.9; 7.3]	**6.5** [4.5; 11.3]	0.34	0.57
medial temporal gyrus	14	27	**4.5** [3.3; 5.4]	5.4 [3.2; 10.9]	0.36	0.57
occipital gyrus	24	37	2.7 [1.7; 4.8]	3.5 [1.9; 8.5]	0.34	0.57
occipital sulcus	24	37	3.2 [2.3; 5]	4.5 [2.4; 9.7]	0.34	0.57
insula cortex	4	27	1.5 [1; 2.1]	5.3 [4; 8.5]	0.34	0.57
claustrum	4	27	0.7 [0.4; 0.9]	2.1 [1; 4]	0.34	0.57
putamen	4	28	0.7 [0.4; 0.8]	1.5 [0.8; 4.5]	0.36	0.57
hippocampus CA1	17	39	0.9 [0.6; 1.8]	1.2 [0.2; 2.7]	0.44	0.57
hippocampus CA2	15	36	0.2 [0; 0.5]	0.2 [0; 0.8]	0.44	0.57
hippocampus CA3	17	35	0.6 [0; 1.5]	1.1 [0; 2.6]	0.36	0.57
hippocampus CA4	17	39	1.2 [0.2; 1.5]	1.1 [0.4; 2.5]	0.92	0.99
subiculum	17	39	1.2 [0.6; 3.3]	2.2 [1.1; 5.2]	0.39	0.57
parahippocampal gyrus	15	38	1.6 [0.6; 2.9]	3.6 [1; 5]	0.34	0.57
thalamus medial nucl.	6	19	0.4 [0.3; 1.3]	1.3 [0.1; 3.6]	0.44	0.57
thalamus ventrolat. nucl.	6	19	0.3 [0.2; 1.3]	1.2 [0.1; 2.1]	0.44	0.57
cerebellar cortex	24	35	0.8 [0.1; 1.4]	0.6 [0; 1.9]	0.44	0.57
cerebellum dentate nucl.	24	35	0 [0; 0.3]	0 [0; 0.6]	0.44	0.57
substantia nigra	24	28	0.8 [0.3; 1.5]	0.6 [0.3; 1.6]	0.81	0.99
locus coeruleus	2	4	0.1 [0; 0.1]	0.8 [0.6; 1.1]	0.44	0.99
amygdala	8	16	2.5 [1.5; 2.9]	1.8 [0.6; 3.5]	0.98	0.99
entorhinal gyrus	8	15	2.1 [1.5; 3.5]	1.5 [1; 2.1]	0.65	0.76
olfactory bulb	0	1	nan [nan; nan]	2.8 [2.8; 2.8]	nan	nan

All p-values were FDR corrected. The three highest median Aβ loads of αSyn+ and αSyn- groups were labeled in bold. *cor.* corrected, *FDR* false discovery rate, *IQR* interquartile range, *nan* not enough values available, *p* p-value, *uncor.* Uncorrected

Table S8 *Available n, median and interquartile range of α-syn covered area of α-syn subgroups*

Region cluster	n (αSyn+A)	Median [IQR] [%] of αSyn+A	n (αSyn+B)	Median [IQR] [%] of αSyn+B	n (αSyn+C)	Median [IQR] [%] of αSyn+C
cortical	108	0.0038 [0.0011; 0.017]	45	0.067 [0.022; 0.12]	186	0.43 [0.10; 1.44]

subcortical	34	0.005 [0.0011; 0.020]	6	0.059 [0.050; 0.11]	53	0.31 [0.12; 0.58]
hippocampal	59	0.014 [0.0026; 0.13]	24	0.062 [0.020; 0.24]	87	0.58 [0.19; 1.11]
amygdala-entorhinal	28	**0.47** [0.118; 0.86]	7	0.088 [0.044; 0.38]	33	**1.28** [0.58; 3.24]
brainstem	26	0.0076 [0.0039; 0.091]	9	**0.643** [0.35; 1.078]	37	0.41 [0.092; 1.09]

The highest median α-syn covered area of each subgroup was labeled in bold. *IQR* interquartile range

Table S9 *Available n, median and interquartile range of tau covered area of α-syn subgroups*

Region cluster	n (αSyn+A)	Median [IQR] [%] of αSyn+A	n (αSyn+B)	Median [IQR] [%] of αSyn+B	n (αSyn+C)	Median [IQR] [%] of αSyn+C
cortical	120	17.6 [9.9; 24.6]	44	4.0 [0.5; 10.9]	186	12.6 [4.4; 23.4]
subcortical	32	1.6 [0.6; 5.2]	6	1.0 [0.3; 2.5]	32	1.9 [0.5; 6.1]
hippocampal	81	18.9 [10.4; 27.2]	30	9.6 [3.2; 15.1]	105	**20.8** [13.7; 28.6]
amygdala-entorhinal	20	**20.6** [14.9; 30.1]	5	**11.1** [9.9; 17.2]	36	20.5 [12.1; 31.4]
brainstem	21	1.9 [1.1; 2.6]	7	1.3 [0.9; 1.8]	27	2.1 [1.5; 3.1]

The highest median tau covered area of each subgroup was labeled in bold. *IQR* interquartile range

Table S10 *Available n, median and interquartile range of Aβ covered area of α-syn subgroups*

Region cluster	n (αSyn+A)	Median [IQR] [%] of αSyn+A	n (αSyn+B)	Median [IQR] [%] of αSyn+B	n (αSyn+C)	Median [IQR] [%] of αSyn+C
cortical	97	**4.7** [3.4; 9.4]	30	**4.0** [2.2; 5.8]	152	**6.1** [3.0; 10.3]
subcortical	34	1.9 [1.0; 3.8]	10	0.8 [0.4; 1.8]	47	1.3 [0.2; 2.8]
hippocampal	81	1.4 [0.4; 3.7]	22	0.5 [0.0; 1.9]	117	1.2 [0.3; 3.3]
amygdala-entorhinal	8	0.9 [0.5; 1.5]	6	1.5 [0.6; 2.0]	15	2.0 [1.2; 2.9]
brainstem	10	1.0 [0.4; 1.7]	5	0.5 [0.5; 0.6]	17	0.8 [0.1; 2.0]

The highest median Aβ covered area of each subgroup was labeled in bold. *IQR* interquartile range

Table S11 *Comparison of αSyn- cases (-) vs. α-syn positive subgroups.*

Subgroups are defined as αSyn+A (+A), amygdala predominant, αSyn+B (+B), brainstem predominant, and αSyn+C (+C), cortical α-syn. These subgroups were compared pairwise with multiple linear regression correcting only for the specific brain region names (uncor.), correcting additionally for age and sex or as a linear mixed effects model with an additional random factor for the subject ID. Effect size (β) and p-value are presented, respectively. Columns A and B indicate which pairs were compared in each row.

Region cluster	A	B	α-syn β, p, uncor.	α-syn β, p, age, sex cor.	α-syn β, p, age, sex, ID cor.	tau β, p, uncor.	tau β, p, age, sex cor.	tau β, p, age, sex, ID cor.	Aβ β, p, uncor.	Aβ β, p, age, sex cor.	Aβ β, p, age, sex, ID cor.
cortical	-	+A	β<0.001, **p=0.047**	β<0.001, **p=0.007**	β<0.001, p=0.22	β=0.02, p=0.1	β=0.031, **p=0.004**	β=0.02, p=0.47	β=0.019, **p=0.014**	β=0.018, **p=0.037**	β=0.017, p=0.77
cortical	-	+B	β<0.001, **p<0.001**	β<0.001, **p<0.001**	β<0.001, **p<0.001**	β=-0.037, **p<0.001**	β=-0.035, **p<0.001**	β=-0.038, p=0.09	β=-0.003, p=0.59	β=-0.002, p=0.77	β=-0.003, p=0.91
cortical	-	+C	β=0.006, **p<0.001**	β=0.006, **p<0.001**	β=0.006, p=0.05	β=-0.003, p=0.43	β=-0.008, **p=0.022**	β=-0.008, p=0.42	β=0.009, **p<0.001**	β=0.007, **p=0.01**	β=0.005, p=0.77

cortical	+ A	+ B	β=0.001, **p<0.001**	β=0.001, **p=0.004**	β=0.001, p=0.09	β=-0.095, **p<0.001**	β=-0.109, **p<0.001**	β=-0.106, p=0.07	β=-0.025, p=0.09	β=-0.024, p=0.19	β=-0.024, p=0.77
cortical	+ A	+ C	β=0.008, **p<0.001**	β=0.01, **p<0.001**	β=0.01, **p=0.022**	β=-0.015, **p=0.037**	β=-0.024, **p<0.001**	β=-0.018, p=0.41	β=0.004, p=0.44	β=0.001, p=0.82	β>-0.001, p=1
cortical	+ B	+ C	β=0.015, **p=0.007**	β=0.016, **p=0.004**	β=0.015, p=0.25	β=0.06, **p=0.001**	β=0.034, p=0.11	β=0.046, p=0.42	β=0.03, **p=0.036**	β=0.018, p=0.34	β=0.018, p=0.77
subcortical	-	+ A	β<0.001, p=0.16	β<0.001, p=0.06	β<0.001, p=0.09	β=0.002, p=0.75	β=0.005, p=0.6	β=0.004, p=0.73	β=0.022, **p=0.025**	β=0.022, **p=0.048**	β=0.019, p=0.77
subcortical	-	+ B	β<0.001, **p<0.001**	β<0.001, **p<0.001**	β<0.001, **p<0.001**	β=-0.007, p=0.37	β=-0.009, p=0.26	β=-0.008, p=0.42	β=0.003, p=0.35	β=0.004, p=0.34	β=0.004, p=0.77
subcortical	-	+ C	β=0.002, **p=0.005**	β=0.002, **p=0.006**	β=0.002, **p=0.022**	β=0.002, p=0.66	β=-0.004, p=0.34	β=-0.004, p=0.47	β=0.004, p=0.24	β=0.002, p=0.57	β=0.002, p=0.81
subcortical	+ A	+ B	β<0.001, p=0.21	β<0.001, p=0.66	β<0.001, p=0.63	β=-0.018, p=0.37	β=-0.024, p=0.22	β=-0.024, p=0.24	β=-0.016, p=0.29	β=-0.012, p=0.48	β=-0.006, p=0.91
subcortical	+ A	+ C	β=0.002, **p=0.002**	β=0.002, **p=0.004**	β=0.002, **p=0.021**	β=0.002, p=0.75	β=-0.01, p=0.26	β=-0.008, p=0.42	β=-0.004, p=0.33	β=-0.005, p=0.34	β=-0.004, p=0.77
subcortical	+ B	+ C	β=0.006, p=0.12	β=0.005, p=0.15	β=0.005, p=0.19	β=0.02, p=0.5	β=0.003, p=0.94	β=0.003, p=0.95	β=0.004, p=0.67	β=-0.007, p=0.58	β=-0.007, p=0.82
hippocampal	-	+ A	β=0.001, **p=0.005**	β=0.001, **p=0.012**	β=0.001, p=0.11	β=-0.012, p=0.45	β=-0.009, p=0.6	β=-0.008, p=0.84	β=0.014, **p=0.025**	β=0.012, p=0.09	β=0.012, p=0.77
hippocampal	-	+ B	β=0.001, **p=0.005**	β=0.002, **p<0.001**	β=0.002, **p<0.001**	β=-0.048, **p<0.001**	β=-0.047, **p<0.001**	β=-0.046, p=0.07	β=-0.001, p=0.61	β>-0.001, p=0.97	β=0.001, p=0.94
hippocampal	-	+ C	β=0.003, **p<0.001**	β=0.003, **p<0.001**	β=0.003, **p=0.002**	β=0.001, p=0.75	β=-0.001, p=0.88	β>-0.001, p=0.97	β=0.002, p=0.08	β=0.001, p=0.48	β=0.001, p=0.87
hippocampal	+ A	+ B	β=0.001, p=0.17	β=0.001, p=0.06	β=0.001, p=0.16	β=-0.084, **p=0.001**	β=-0.1, **p<0.001**	β=-0.1, p=0.12	β=-0.017, p=0.24	β=-0.012, p=0.48	β=-0.01, p=0.91
hippocampal	+ A	+ C	β=0.004, **p<0.001**	β=0.004, **p<0.001**	β=0.004, **p<0.001**	β=0.008, p=0.37	β=0.005, p=0.6	β=0.005, p=0.76	β=-0.003, p=0.32	β=-0.005, p=0.3	β=-0.005, p=0.77
hippocampal	+ B	+ C	β=0.007, **p=0.005**	β=0.008, **p=0.004**	β=0.008, p=0.022	β=0.1, **p<0.001**	β=0.1, **p<0.001**	β=0.1, **p=0.014**	β=0.01, p=0.24	β=0.005, p=0.57	β=0.003, p=0.91
amygdala-entorhinal	-	+ A	β=0.008, **p<0.001**	β=0.007, **p=0.001**	β=0.007, **p=0.001**	β=-0.031, p=0.37	β=-0.001, p=0.98	β=-0.001, p=0.98	β=-0.013, p=0.24	β=-0.012, p=0.34	β=-0.012, p=0.77
amygdala-entorhinal	-	+ B	β=0.001, **p<0.001**	β=0.001, **p<0.001**	β=0.001, **p<0.001**	β=-0.059, p=0.07	β=-0.066, **p=0.021**	β=-0.069, p=0.09	β=-0.006, p=0.34	β=-0.004, p=0.48	β=-0.004, p=0.77
amygdala-entorhinal	-	+ C	β=0.007, **p<0.001**	β=0.008, **p<0.001**	β=0.008, **p<0.001**	β=-0.013, p=0.22	β=-0.016, p=0.08	β=-0.015, p=0.24	β<0.001, p=0.95	β=0.001, p=0.82	β=0.001, p=0.91
amygdala-entorhinal	+ A	+ B	β=-0.006, p=0.24	β=-0.003, p=0.66	β=-0.003, p=0.63	β=-0.091, p=0.18	β=-0.175, p=0.06	β=-0.181, p=0.12	β=0.002, p=0.83	β=0.003, p=0.82	β=0.003, p=0.91
amygdala-entorhinal	+ A	+ C	β=0.007, **p=0.014**	β=0.009, **p=0.004**	β=0.008, **p=0.017**	β=-0.004, p=0.75	β=-0.018, p=0.31	β=-0.018, p=0.45	β=0.007, p=0.32	β=0.01, p=0.34	β=0.01, p=0.77
amygdala-entorhinal	+ B	+ C	β=0.02, **p=0.046**	β=0.022, **p=0.032**	β=0.022, p=0.06	β=0.08, p=0.23	β=0.09, p=0.16	β=0.1, p=0.25	β=0.012, p=0.39	β=0.014, p=0.48	β=0.014, p=0.77
brainstem	-	+ A	β<0.001, **p=0.008**	β=0.001, **p=0.005**	β=0.001, **p=0.021**	β=-0.007, p=0.33	β=-0.005, p=0.46	β=-0.005, p=0.54	β=0.002, p=0.61	β=0.003, p=0.58	β=0.004, p=1
brainstem	-	+ B	β=0.004, **p<0.001**	β=0.004, **p<0.001**	β=0.004, **p<0.001**	β=-0.007, p=0.18	β=-0.009, p=0.06	β=-0.008, p=0.2	β=-0.003, p=0.35	β=-0.003, p=0.48	β=nan, p=nan
brainstem	-	+ C	β=0.003, **p=0.001**	β=0.003, **p=0.002**	β=0.003, p=0.022	β=0.001, p=0.74	β=0.001, p=0.74	β=0.001, p=0.73	β=0.001, p=0.46	β=-0.001, p=0.71	β=-0.001, p=0.87
brainstem	+ A	+ B	β=0.007, **p<0.001**	β=0.007, **p<0.001**	β=0.007, **p<0.001**	β=-0.008, p=0.37	β=-0.011, p=0.26	β=-0.009, p=0.42	β=-0.008, p=0.27	β=-0.009, p=0.34	β=-0.009, p=1
brainstem	+ A	+ C	β=0.004, **p=0.008**	β=0.004, **p=0.005**	β=0.005, **p=0.049**	β=0.005, p=0.33	β=0.003, p=0.58	β=0.003, p=0.54	β=0.001, p=0.83	β=-0.003, p=0.52	β=-0.003, p=0.77
brainstem	+ B	+ C	β<0.001, p=0.98	β<0.001, p=0.93	β=0.003, p=0.72	β=0.017, p=0.28	β=0.014, p=0.39	β=0.014, p=0.42	β=0.008, p=0.37	β=0.002, p=0.82	β=0.002, p=0.91

All p-values were FDR corrected. Significant p-values were labeled in bold. *cor.* corrected, *FDR* false discovery rate, *ID* subject ID, *nan* not enough values available, *p* p-value, *uncor.* uncorrected

Supplementary Figures

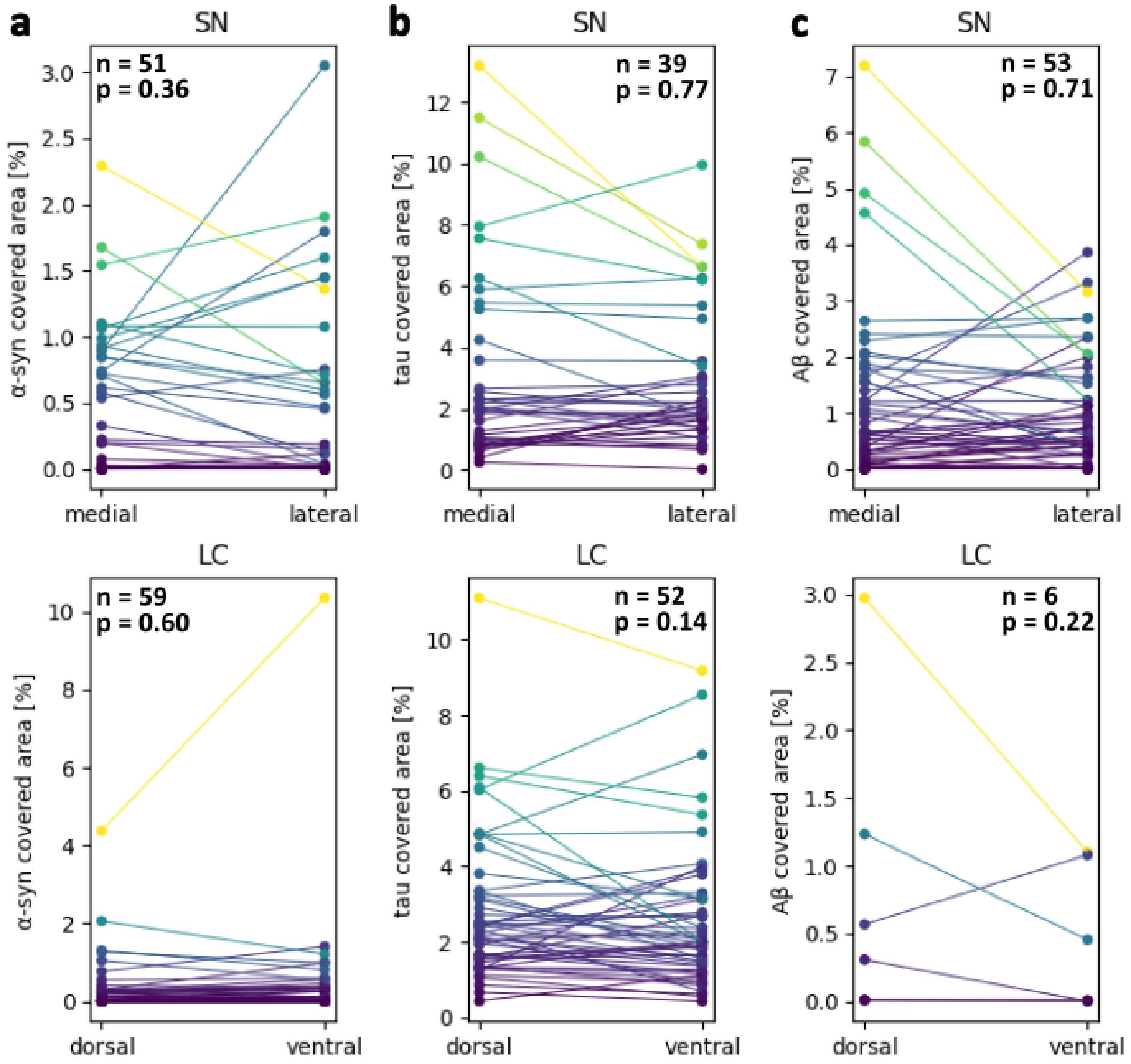

Fig. S1 *Deposit quantification in the substantia nigra (SN) and locus coeruleus (LC).*

(a) α-synuclein, (b) tau, and (c) Aβ. A more medial vs. a more lateral representative area was manually annotated in the SN and a dorsal vs. a more ventral area in the LC, avoiding pigmented neurons, respectively. This approach may introduce a bias towards more affected parts; however, it prevents false positive classifications. Here, we assess the extent to which we were successful in reproducibly selecting representative areas by comparing the covered areas of the two markings sorted by rough location. Despite some variability, there were no significant differences, as tested with a Wilcoxon signed-rank test, supporting the reliability of the annotations. Since the annotations were not explicitly selected for a comparison of the different localizations, the significance in this direction is limited. LC locus coeruleus, SN substantia nigra

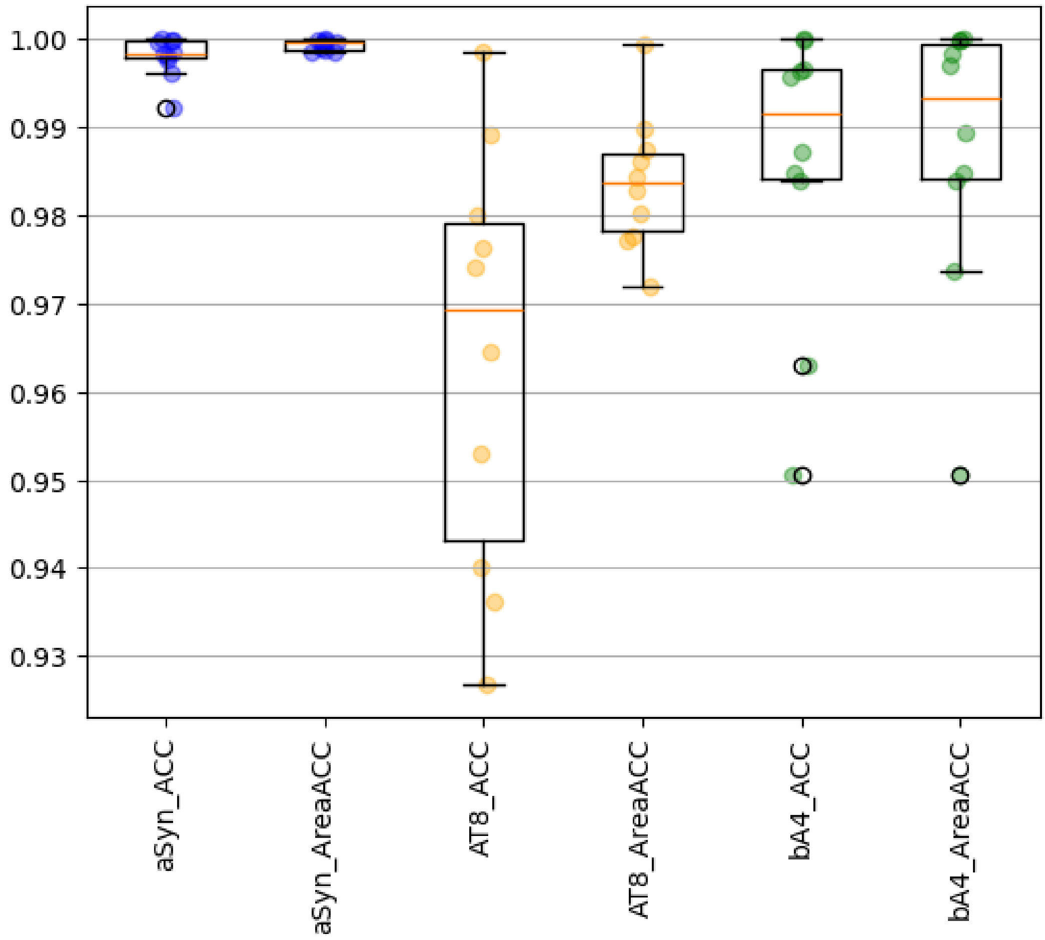

Fig. S2 *Accuracy of the ilastik random forest pixel classifiers tested with ten test images.*

For each staining, namely alpha-Synuclein (aSyn), tau (AT8), and Amyloid β (bA4), the accuracy was calculated as follows: ACC = (true positive pixels + true negative pixels) / all pixels. Thus, a value close to 1 is a perfect match. As an additional index, the area-accuracy was measured as an accuracy of the absolute pixel values independent of the pixel localization: AreaACC = 1 - (|Ground truth covered area - predicted covered area| / all pixels)

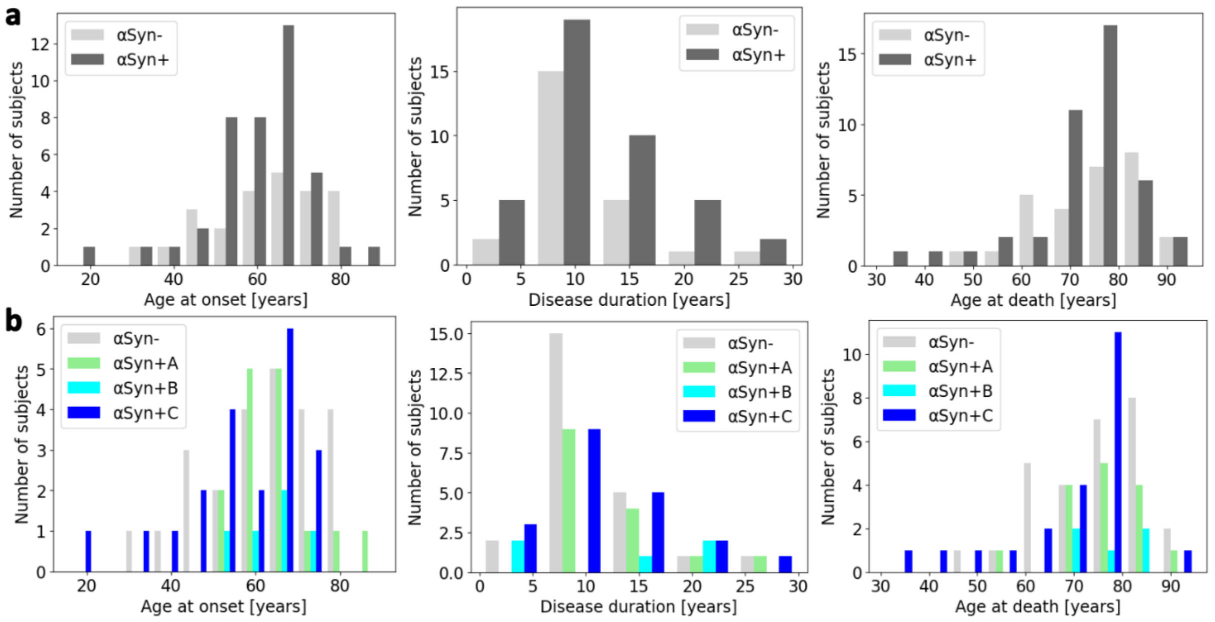

Fig. S3 *Age at clinically reported symptom onset, disease duration, and age at death of Alzheimer's disease patients with and without Lewy body co-pathology.*

(a) Histograms comparing clinical data of αSyn- and αSyn+ groups. (b) Histograms comparing clinical data of αSyn- and αSyn+A (amygdala predominant), αSyn+B (brainstem predominant), αSyn+C (cortical) α-syn positive subgroups.

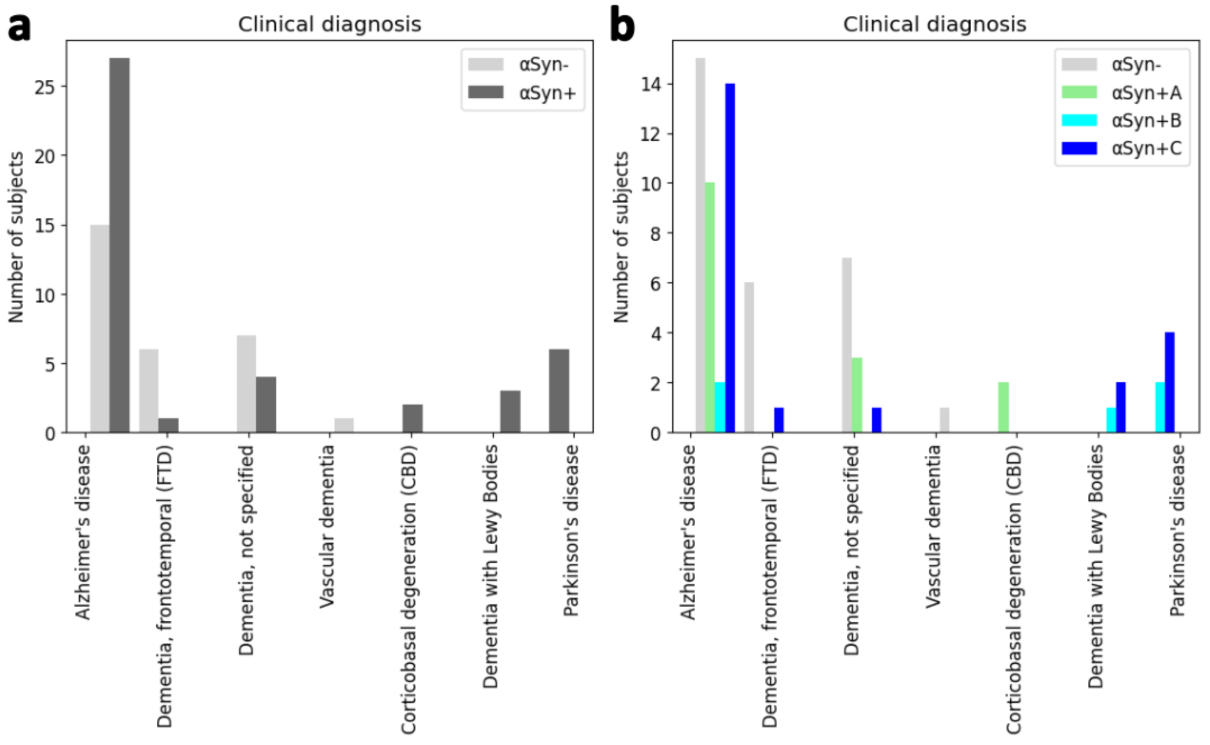

Fig. S4 *Clinical diagnoses of patients with neuropathologically confirmed extensive Alzheimer's disease related neuropathological change.*

The majority of cases was already clinically diagnosed with Alzheimer's disease. (a) Histogram presenting clinical diagnoses of αSyn- and αSyn+ cases. (b) Histogram presenting clinical diagnoses of

αSyn- and αSyn+A (amygdala predominant), αSyn+B (brainstem predominant), αSyn+C (cortical) α-syn positive subgroups.

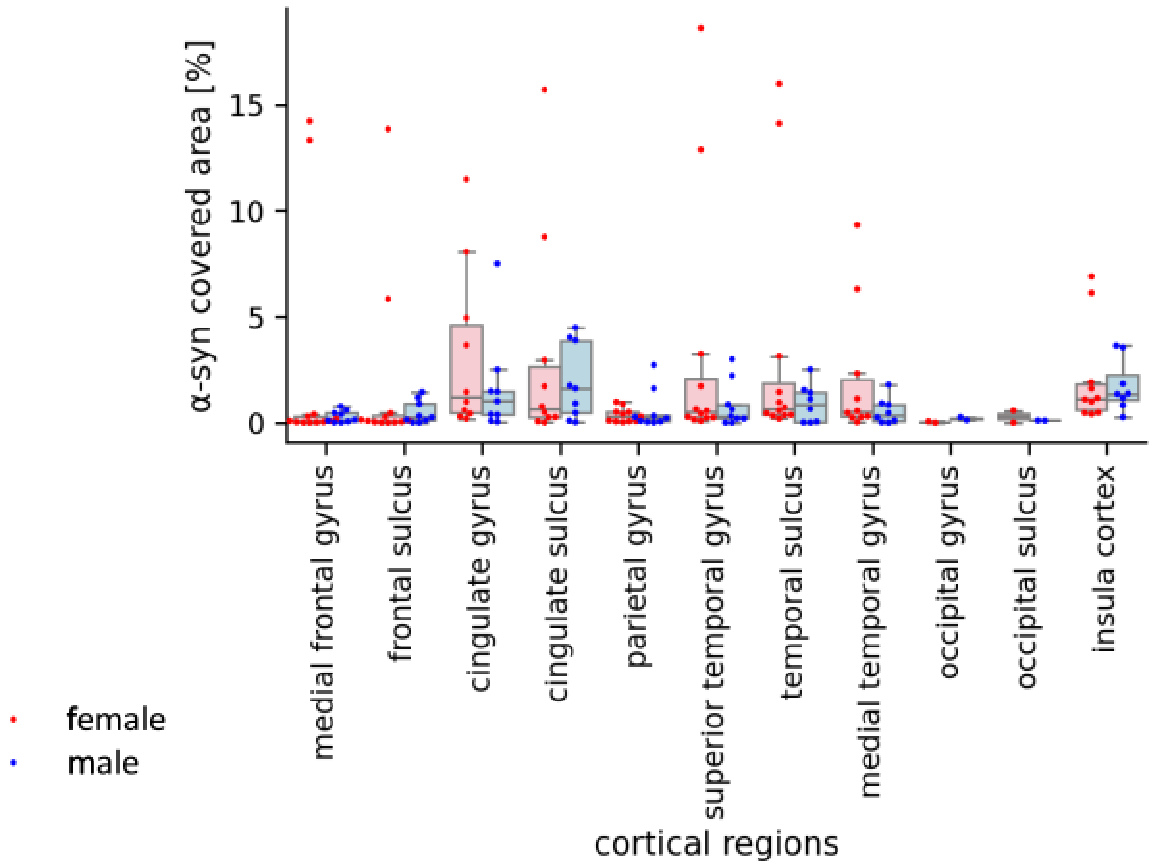

Fig. S5 *Boxplots comparing cortical α-synuclein (α-syn) deposit loads in female vs. male Alzheimer's disease (AD) patients with cortical Lewy pathology (αSyn+C).*

In a region-wise comparison, the α-syn load is comparable between sexes. The female group shows more outliers with high α-syn loads which added up to a slight trend of higher loads in females with a multiple linear regression model but is not significant after false discovery rate correction and seems to be mainly driven by individuals. In summary, we cannot identify a distinct sex difference of cortical α-syn covered area in AD with cortical Lewy pathology.

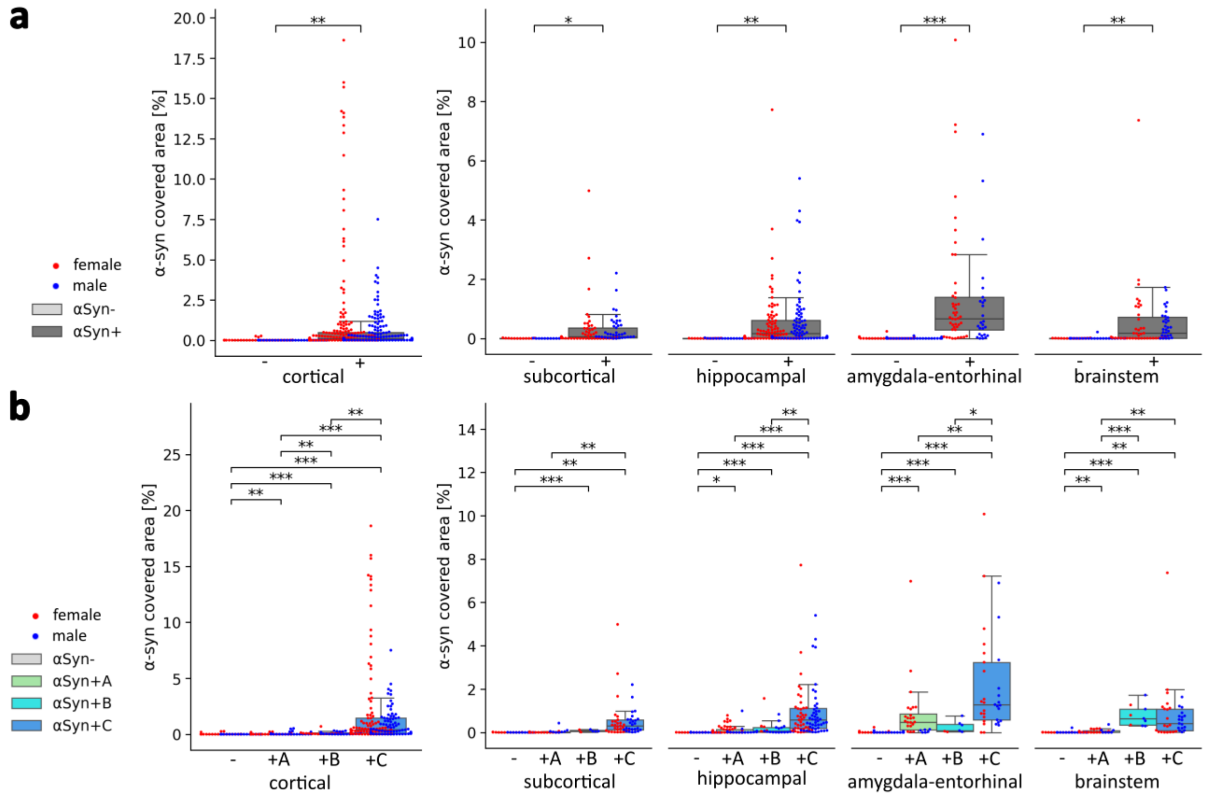

Fig. S6 *Alpha-synuclein (α-syn) load and distribution in Alzheimer's disease cases.*

This Figure equals Fig. 2b and c of the main manuscript with an additional layer of scatter dots. Each dot represents one analyzed brain region of one patient. Regions from female patients are shown in red, regions from male patients in blue, i.e., one patient is represented by several cortical dots, several subcortical dots, etc. (a) Comparison of the α-syn covered area between αSyn- and αSyn+ groups. (b) Comparison of the α-syn covered area between αSyn- and αSyn+A (amygdala predominant), αSyn+B (brainstem predominant), αSyn+C (cortical) α-syn positive subgroups. Statistics in (a) and (b) were calculated with multiple linear regression across region clusters, controlling for region names, age, and sex, and false discovery rate correction.

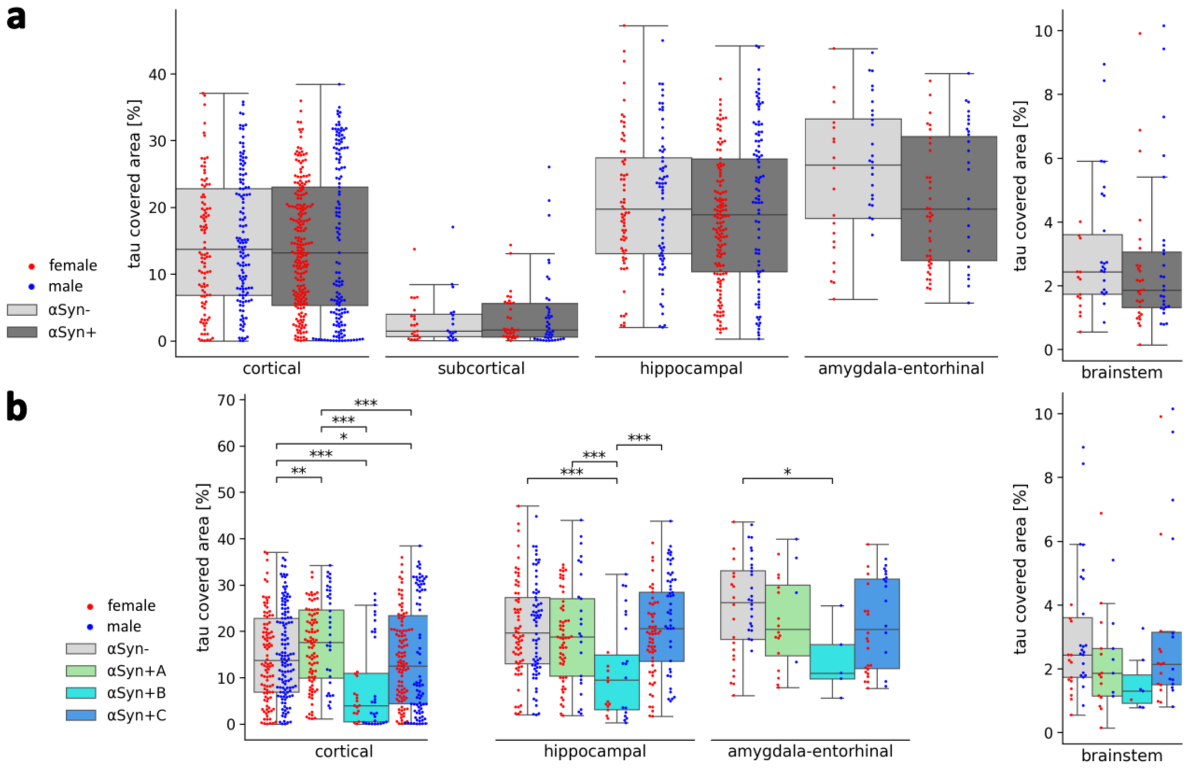

Fig. S7 *Tau load and distribution in Alzheimer's disease.*

This Figure equals Fig. 3b and c of the main manuscript with an additional layer of scatter dots. Each dot represents one analyzed brain region of one patient. Regions from female patients are shown in red, regions from male patients in blue, i.e., one patient is represented by several cortical dots, several subcortical dots, etc. (a) Comparison of the tau covered area between αSyn- and αSyn+ groups. (b) Comparison of the tau covered area between αSyn- and αSyn+A (amygdala predominant), αSyn+B (brainstem predominant), αSyn+C (cortical) α-syn positive subgroups. Statistics in (a) and (b) were calculated with multiple linear regression across region clusters, controlling for region names, age, and sex, and false discovery rate correction.

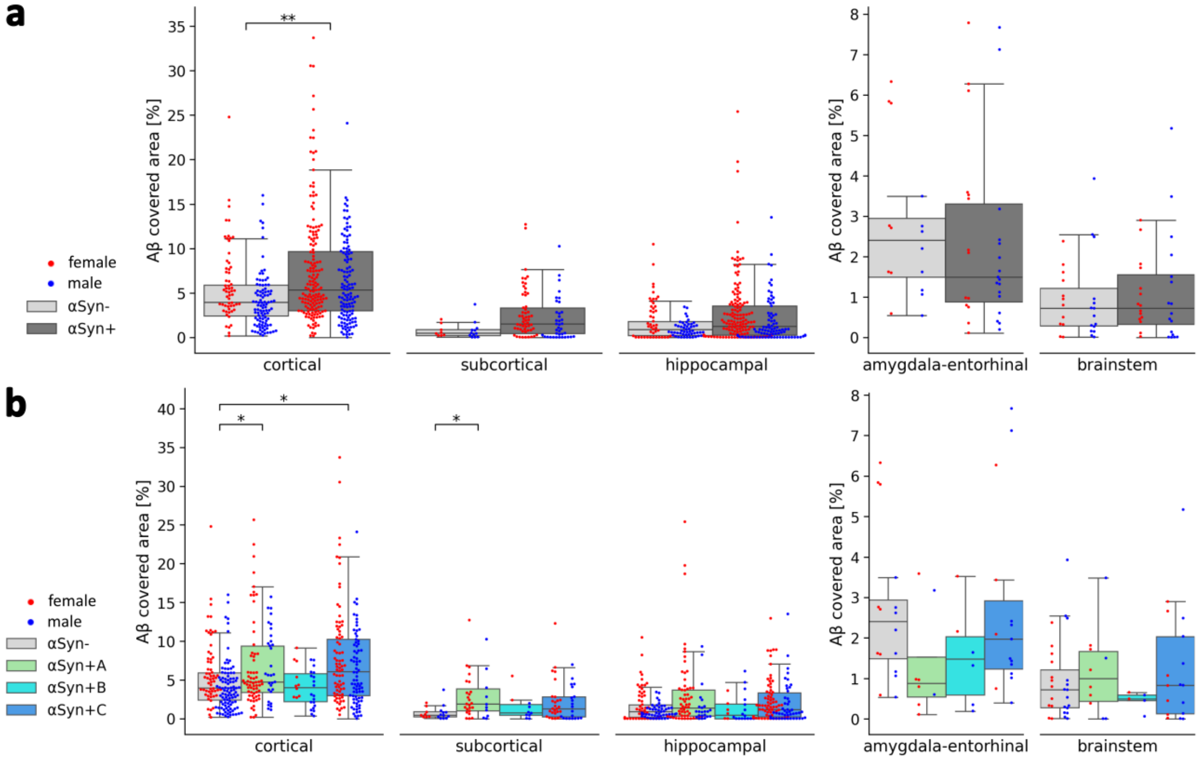

Fig. S8 *Amyloid beta (Aβ) load and distribution in Alzheimer's disease.*

This Figure equals Fig. 4b and c of the main manuscript with an additional layer of scatter dots. Each dot represents one analyzed brain region of one patient. Regions from female patients are shown in red, regions from male patients in blue, i.e., one patient is represented by several cortical dots, several subcortical dots, etc. (a) Comparison of the Aβ covered area between αSyn- and αSyn+ groups. (b) Comparison of the Aβ covered area between αSyn- and αSyn+A (amygdala predominant), αSyn+B (brainstem predominant), αSyn+C (cortical) α-syn positive subgroups. Statistics in (a) and (b) were calculated with multiple linear regression across region clusters, controlling for region names, age, and sex, and false discovery rate correction.

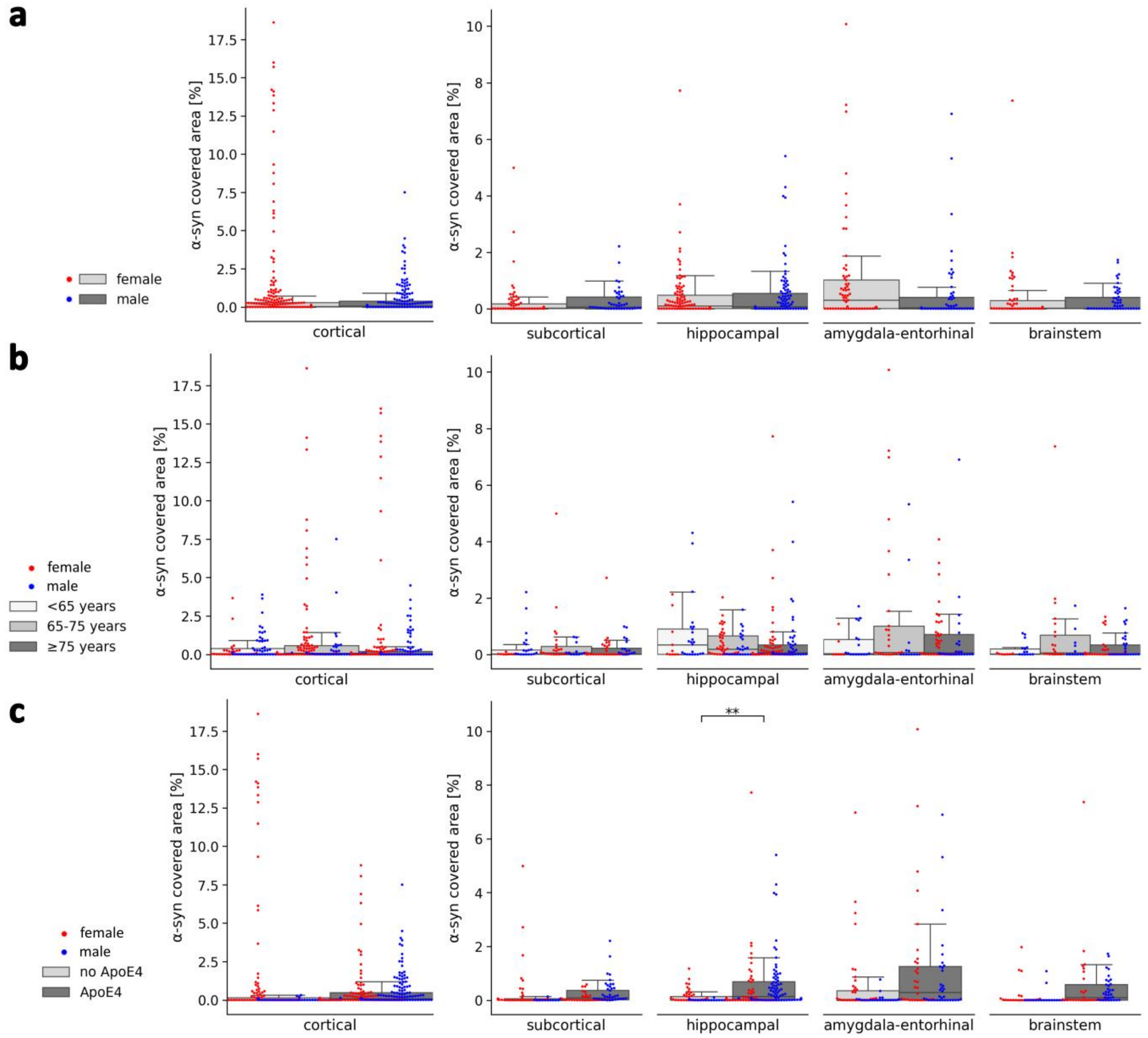

Fig. S9 *α-Syn load split up by (a) sex, (b) age at death, and (c) ApoE genotype in Alzheimer's disease cases.*

This Figure equals Fig. 5 of the main manuscript with an additional layer of scatter dots. Each dot represents one analyzed brain region of one patient. Regions from female patients are shown in red, regions from male patients in blue, i.e., one patient is represented by several cortical dots, several subcortical dots, etc. Statistics were calculated with multiple linear regression across region clusters, correcting for specific region names and false discovery rate correction. Results with age or sex correction are presented in the main text. ApoE4 means that at least one ApoE4 allele is apparent.

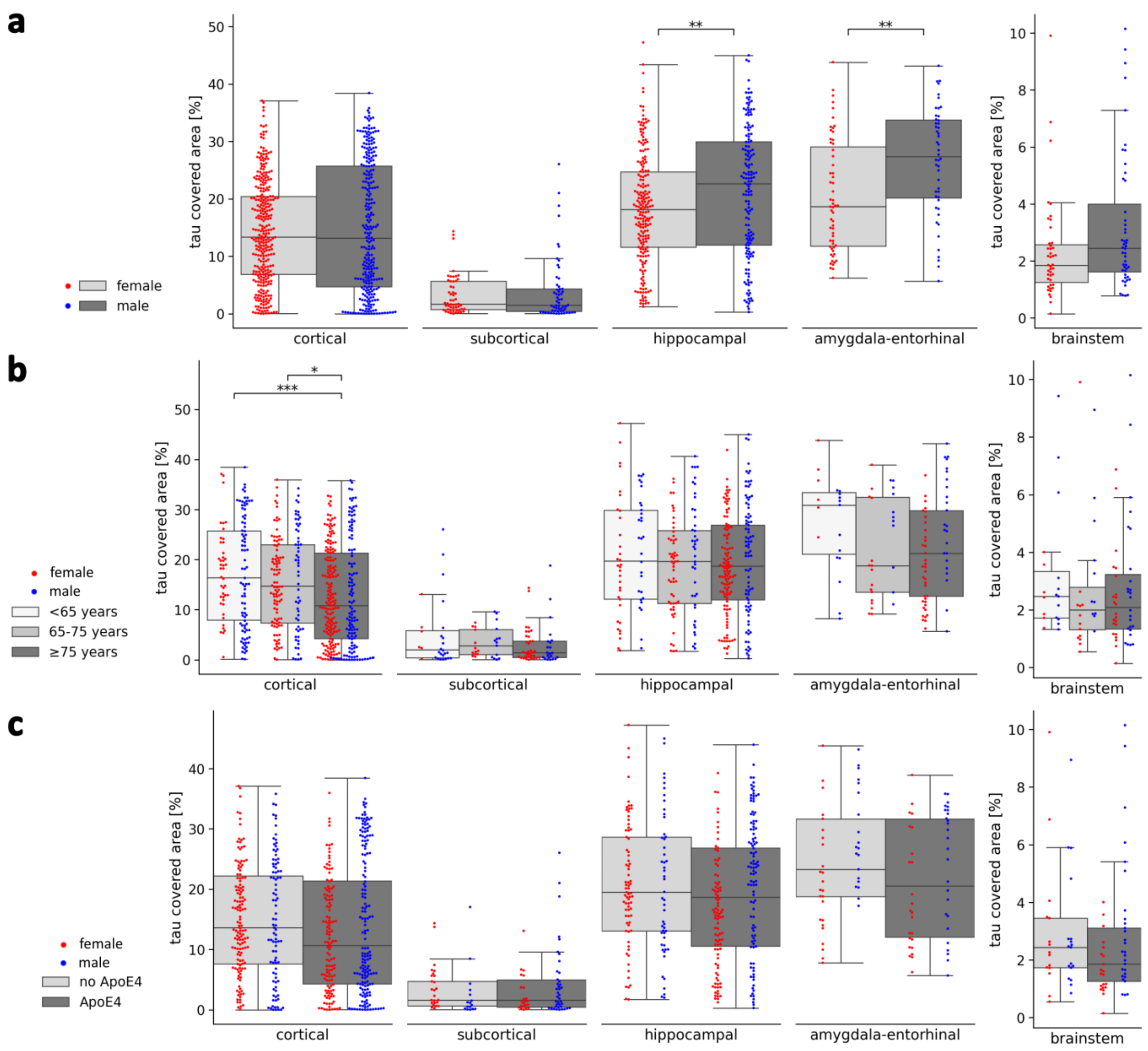

Fig. S10 *Tau load split up by (a) sex, (b) age at death and (c) ApoE genotype in Alzheimer's disease cases.*

Each dot represents one analyzed brain region of one patient. Regions from female patients are shown in red, regions from male patients in blue, i.e., one patient is represented by several cortical dots, several subcortical dots, etc. Statistics were calculated with multiple linear regression across region clusters correcting for specific region names and with false discovery rate correction but without age or sex correction. ApoE4 means that at least one ApoE4 allele is apparent.

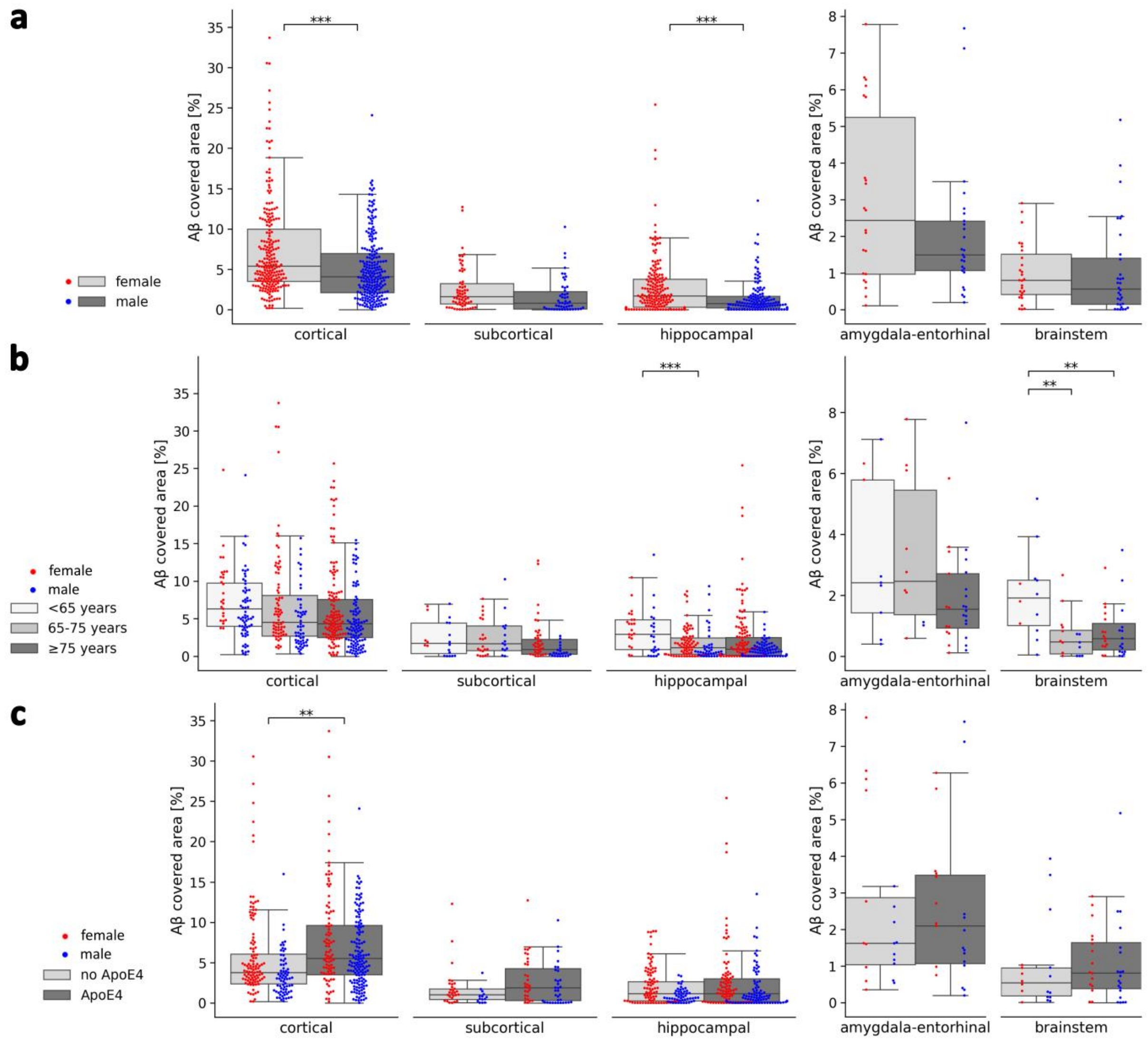

***Fig. S11** Aβ load split up by (a) sex, (b) age at death and (c) ApoE genotype in Alzheimer's disease cases.*

Each dot represents one analyzed brain region of one patient. Regions from female patients are shown in red, regions from male patients in blue, i.e., one patient is represented by several cortical dots, several subcortical dots, etc. Statistics were calculated with multiple linear regression across region clusters correcting for specific region names and with false discovery rate correction but without age or sex correction. ApoE4 means that at least one ApoE4 allele is apparent.

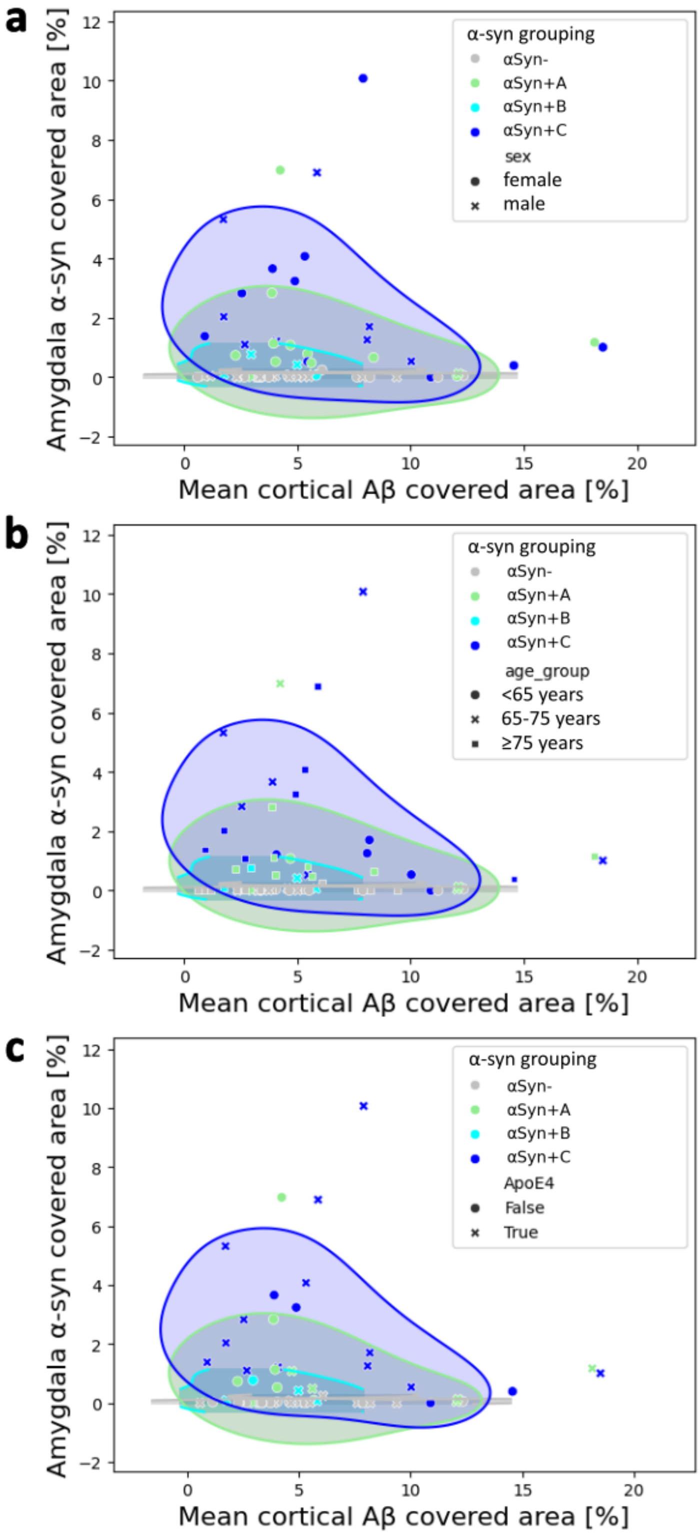

***Fig. S12** Overview of cortical Aβ load vs. α-syn load in the amygdala.*

Scatter plots showing the mean cortical Aβ load together with the α-syn covered area in the amygdala for αSyn- and αSyn+ cases. Every dot represents one case with additional encoding of (a) sex, (b) age at death, and (c) ApoE4 carriage. Density curves were created with a seaborn kernel density estimate (KDE) plot in Python (Python version 3.10.12) for easier interpretation.
